# Leveraging human-centered design and causal pathway diagramming toward enhanced specification and development of innovative implementation strategies: a case example of an outreach tool to address racial inequities in breast cancer screening

**DOI:** 10.1186/s43058-024-00569-w

**Published:** 2024-03-28

**Authors:** Leah M. Marcotte, Raina Langevin, Bridgette H. Hempstead, Anisha Ganguly, Aaron R. Lyon, Bryan J. Weiner, Nkem Akinsoto, Paula L. Houston, Victoria Fang, Gary Hsieh

**Affiliations:** 1 Department of Medicine, University of Washington School of Medicine, 908 Jefferson St, Seattle, WA 98104 USA; 2 Department of Human Centered Design and Engineering, University of Washington, Seattle, WA USA; 3 Cierra Sisters, Seattle, WA USA; 4 Department of Medicine, University of Texas Southwestern Medical Center, Dallas, TX USA; 5 Department of Psychiatry and Behavioral Sciences, University of Washington School of Medicine, Seattle, WA USA; 6 Departments of Global Health, University of Washington School of Medicine, Seattle, WA USA; 7 Health Systems and Population Health, University of Washington School of Medicine, Seattle, WA USA; 8 UW Medicine, enterprise health system of the University of Washington, Seattle, USA

**Keywords:** Human-centered design, Implementation strategies, Causal pathway diagrams, Healthcare equity

## Abstract

**Background:**

Implementation strategies are strategies to improve uptake of evidence-based practices or interventions and are essential to implementation science. Developing or tailoring implementation strategies may benefit from integrating approaches from other disciplines; yet current guidance on how to effectively incorporate methods from other disciplines to develop and refine innovative implementation strategies is limited. We describe an approach that combines community-engaged methods, human-centered design (HCD) methods, and causal pathway diagramming (CPD)—an implementation science tool to map an implementation strategy as it is intended to work—to develop innovative implementation strategies.

**Methods:**

We use a case example of developing a conversational agent or chatbot to address racial inequities in breast cancer screening via mammography. With an interdisciplinary team including community members and operational leaders, we conducted a rapid evidence review and elicited qualitative data through interviews and focus groups using HCD methods to identify and prioritize key determinants (facilitators and barriers) of the evidence-based intervention (breast cancer screening) and the implementation strategy (chatbot). We developed a CPD using key determinants and proposed strategy mechanisms and proximal outcomes based in conceptual frameworks.

**Results:**

We identified key determinants for breast cancer screening and for the chatbot implementation strategy. Mistrust was a key barrier to both completing breast cancer screening and using the chatbot. We focused design for the initial chatbot interaction to engender trust and developed a CPD to guide chatbot development. We used the persuasive health message framework and conceptual frameworks about trust from marketing and artificial intelligence disciplines. We developed a CPD for the initial interaction with the chatbot with engagement as a mechanism to use and trust as a proximal outcome leading to further engagement with the chatbot.

**Conclusions:**

The use of interdisciplinary methods is core to implementation science. HCD is a particularly synergistic discipline with multiple existing applications of HCD to implementation research. We present an extension of this work and an example of the potential value in an integrated community-engaged approach of HCD and implementation science researchers and methods to combine strengths of both disciplines and develop human-centered implementation strategies rooted in causal perspective and healthcare equity.

**Supplementary Information:**

The online version contains supplementary material available at 10.1186/s43058-024-00569-w.

Contributions to the literature

- The integration of human-centered design and implementation science researchers and methods can synthesize strengths of both disciplines.
- Human-centered design methods can be employed as part of an overarching co-creation approach to including partners/communities in research.
- A human-centered design approach rooted in causal pathway diagramming can help to address challenges of both basing implementation strategies in theory and meeting the needs of partners and/or communities.

## Background

The field of implementation science was created to address the gap between what *should* be done based on existing evidence and what *is* done in practice. Implementation strategies—“methods or techniques used to enhance the adoption, implementation, and sustainability of a clinical program or practice” [1]—are central to implementation science. In 2015, the Expert Recommendations for Implementing Change project compiled 73 different implementation strategies used in the field [2]. However, as implementation science has evolved, experts have recognized that (a) more implementation strategies exist than have been cataloged and (b) developing or tailoring implementation strategies may benefit from integrating approaches from other disciplines (e.g., behavioral economics and human-centered design) [3–5]. Yet, current guidance on how to effectively incorporate methods from other disciplines to develop and refine innovative implementation strategies is limited.

The causal pathway diagram (CPD) is an implementation science method that can be used to support development and refinement of implementation strategies [6]. CPDs help researchers to understand implementation strategies as they are intended to work. In building a CPD, researchers identify the implementation strategy, the mechanism(s) through which the strategy is thought to lead to the intended outcome, proximal outcomes which may provide signals of effect earlier than the intended outcome, and the distal (intended) outcome. CPDs also include moderators which may enhance or dampen pathway effect and pre-conditions which are necessary for the pathway to proceed. In developing and refining implementation strategies, CPDs help investigators map key determinants (barriers or facilitators) that implementation strategies address and mechanisms by which strategies are posited to effect change. Theory and existing evidence are typically used to construct CPDs [7]. One potential limitation of the CPD is that while there is an emphasis on incorporating theory and existing evidence, there are fewer examples of incorporating implementation partners’ and/or community needs and context in the initial creation of the CPD [8].

Co-creation—a collaborative process including people with a diversity of roles/positions to attain goals—is increasingly recognized as an approach for implementation scientists to integrate partners/communities in research [9]. In co-creation, researchers may employ several different methodologies and methods. For example, many researchers use community-engaged research approaches to meaningfully include community members in intervention development and implementation with goals to increase relevance, effectiveness, and sustainment of interventions [9, 10]. Co-creation can be framed as an overarching concept that includes co-design (intervention development) and co-production (intervention implementation) [11]. Co-design is specifically relevant for the design of novel implementation strategies and researchers often use human-centered design methods for co-designing interventions [12, 13]. In co-design, multiple methods may be used synergistically. For example, researchers can simultaneously use community-based participatory research methods to meaningfully include community members and human-centered design (HCD) methods to guide intervention design [14, 15].

HCD methods are particularly useful in co-design for technology-based implementation strategies with key standards and principles for designing interactive systems [16]. HCD is a “flexible, yet disciplined and repeatable approach to innovation that puts people at the center of activity.” [17] HCD methods elicit information regarding user environment and experience through continuous partner/user engagement and draw from multidisciplinary expertise [18]. An initial phase of HCD is to establish the context of use and requirements of users [19]. In this exploratory phase, researchers often collect and analyze qualitative data through interviews, focus groups, and/or co-design sessions. In addition to questions about context and user requirements, interviews will often include “mockups” or early prototypes for initial reactions and feedback.

Establishing context of use and user requirements in HCD is synergistic to the needs for specifying implementation strategies and developing CPDs [20]. HCD methods can be used to build and inform CPDs by gaining understanding of key determinants to the desired program or practice within a specific context and identifying potential facilitators and barriers to the implementation strategy itself. Early qualitative data from HCD methods and identification of key determinants can help to inform use of theory in building CPDs. Finally, HCD methods can further help to understand and test assumptions related to mechanisms of an implementation strategy [20].

Haines et al. described the application of HCD methods in defining context and connecting to evidence-based practices and implementation strategies. [4] We build on this work by describing a way to explicitly include partners (organizational and community) in the co-design of implementation strategies while maintaining a causal basis and perspective. We present an example incorporating HCD methods in CPDs to design an innovative outreach strategy to address inequities in breast cancer screening using mammography among Black women. In this case study, we illustrate how HCD methods were used to (1) identify and prioritize key determinants, (2) select and apply conceptual frameworks, and (3) understand (and design for) strategy mechanisms.

## Methods

### Case example: designing an outreach tool to address breast cancer screening inequities

Inequities in breast cancer mortality among Black people have been recognized for decades and yet persist [21, 22]. These inequities are partly due to later stage breast cancer diagnosis [21]. Regular interval breast cancer screening with mammography aligned with the United States Preventive Services Task Force (USPSTF) guidelines is an evidence-based intervention to improve earlier diagnosis of and mortality from breast cancer [23]. Therefore, addressing breast cancer screening inequities among Black people eligible for screening aligned with guidelines may facilitate early detection and improve breast cancer survival [24–26]. Research to date has demonstrated that Black women experience multiple barriers to breast cancer screening including reduced access to care, mistrust and decreased self-efficacy, fear of diagnosis, prior negative health care experiences, and lack of information regarding breast cancer risk [27–35]. Black women may also not feel included or prioritized in breast cancer screening campaigns [30].

Tailored interventions to improve breast cancer screening among Black women have demonstrated modest effect in improving breast cancer screening rates, yet many of these interventions, such as use of health navigators, are resource intensive and must be repeated annually [36–47]. Mobile technology interventions can be culturally tailored and may address limitations related to cost and time [48, 49]. Mobile technology interventions using short message service (SMS) text are accessible to individuals across a range of sociodemographic factors and have been shown to be effective in primary care behavioral and disease management interventions [50]. Black women have reported SMS text-based breast cancer screening interventions to be accessible and acceptable [51]. SMS text-based interventions are also more accessible than patient portal-based interventions which lack adequate reach due to substantial racial inequities in patient portal use [52, 53]. Mobile health interventions using conversational interfaces such as chatbots via SMS text can act as virtual health navigators providing individualized information about and connecting individuals to healthcare [54]. Prior research has shown chatbots increase levels of trust in web-based information and are easy to use and scale [55, 56].

While the use of health navigators is an established implementation strategy, there are little data on integrating conversational agents in primary care outreach and none that we are aware of that specifically address healthcare inequities in cancer screening [57]. Literature on digital health interventions emphasizes need for careful attention to and planning for implementation to optimize integration in the healthcare system and patient use [58]. Moreover, evidence of bias in artificial intelligence raises caution in the design of chatbot interventions [59–61]. We identified chatbots as a promising, innovative implementation strategy to address breast cancer screening inequities; however, one that warrants rigorous methods and community engagement to design and tailor.

In late 2020, we brought together a team of researchers and health system leaders at a large academic medical center to address inequities in breast cancer screening through the design of a chatbot that could facilitate outreach. Breast cancer screening rates in the health system at the time using the National Committee for Quality Assurance Healthcare Effectiveness Data and Information Set measure based on the USPSTF guidelines were 61.5% among Black women compared to 73.3% among White women (internal health system data) [23, 62]. The chatbot implementation strategy was favored as an intervention among interdisciplinary team members because of its innovation and the low resource burden to primary care with better potential for sustainability. Usual care consisted of chart review and telephone outreach by a primary care health navigator and then connection to radiology for mammogram scheduling. The chatbot intervention could be sent to people due for screening and could be configured to schedule a mammogram during the chatbot interaction, expending less resources with greater efficiency. The study protocol was reviewed and determined exempt by the University of Washington Institutional Review Board. This manuscript adheres to the Enhancing the Quality and Transparency of health research (EQUATOR) Better Reporting of Interventions: template for intervention description and replication (TIDieR) checklist and guide and the Consolidated criteria for REporting Qualitative research (COREQ) guidelines [63, 64].

#### Personnel

To approach implementation-focused research questions, interdisciplinary teams of researchers, operational partners, and end-users are advantageous to develop optimized implementation strategies or innovations. Implementation science and human-centered design researchers co-leading efforts (e.g., as PI, Co-PI, or MPIs) can help to support integration of methods and perspectives. In patient-facing interventions—particularly those addressing inequities among marginalized communities—including patient/community partners can center intervention development on patient/community needs, facilitate participant recruitment, help refine study protocol, and support the analyses of collected data [10, 65].

In designing a chatbot for breast cancer screening outreach to address racial inequities, our team included an HCD researcher (G.H.), a primary care physician and early-stage investigator with focus in implementation science (L.M.M.), an HCD PhD candidate (R.L.), and a community-based organization leader (B.H.H.) with expertise in conducting interviews and focus groups for qualitative research and extensive community connections. We received project mentorship from the Optimizing Implementation in Cancer Control (OPTICC) team that includes several leaders and experts in implementation science (e.g., B.J.W., A.R.L.) [66]. We drew input from key health system partners including health care equity leadership (P.L.H.), primary care and population health leadership (N.A., V.F.), and primary care health navigators. We held regular interdisciplinary team meetings, most frequently in the initial stages of innovation design. Throughout the development of the chatbot tool, we sought feedback from community members through interviews, focus groups, and (planned) co-design sessions.

#### Positionality statement

Our team included trainees, researchers, clinicians, and operational leaders at the University of Washington and a community-based organization leader. Previous research interests/experience included communication technologies to promote health and well-being (G.H.) and improving quality and equity in primary care services (L.M.M.). B.H.H. provided health equity expertise; she has led a Seattle-based survivor and support organization for African American women with cancer for over 25 years; in that time, she has collaborated with researchers on over 60 grants. Most of our team identifies as women and several of our team members identify as Black women. Research analysis was conducted primarily by R.L., G.H., and L.M.M. (none of whom are Black/African American); all data analysis/interpretation was reviewed with B.H.H. in bi-weekly research meetings.

### Identify and prioritize key determinants

#### Overview

Key determinant (i.e., facilitators and barriers) identification is critical in implementation strategy development and a first step in creating a CPD. Determinants may be identified initially through evidence review and contextually through qualitative (e.g., interviews) and/or quantitative (e.g., survey) methods. The use of HCD methods can augment identification of key determinants and other components in CPD via mockups and/or early prototypes to elicit feedback on initial design and use. This approach is particularly useful because determinants can be elicited in the context of the implementation strategy—which may help to optimize determinant-strategy matching. We identified and prioritized key determinants through rapid evidence review of breast cancer screening determinants among Black women and HCD methods. We conducted qualitative analysis of semi-structured interviews including a chatbot mockup and focus groups with end-users who were shown an early prototype of the chatbot which was iterated based on qualitative data analysis of the interviews. Interviews and focus groups were led almost entirely by B.H.H., a community member, to provide comfortable environments to share perspectives. Our underlying interpretive framework most closely followed social constructivism; we focused on the content of participant words and experiences with the goal to minimize researcher interpretation [67]. Any question of participant meaning was reviewed with B.H.H.

### Rapid evidence review

#### Objective

Our objective was to identify determinants to breast cancer screening among Black women emergent from recent literature.

### Procedure

We conducted rapid evidence review following established methods described in the National Collaborating Centre for Methods and Tools Rapid Review Guidebook [68]. We defined a research question—“among Black women in the United States, what are determinants (i.e., facilitators and barriers) to breast cancer screening?”, searched for research evidence, critically appraised information sources, and synthesized evidence.

### Search strategy

Our search strategy prioritized evidence in the past 3 years and included search terms in or related to the research question: (Mammogram, Mammography, Cancer Screening, Breast Cancer Screening), (Breast Cancer), (Women), (Black, African American, African American, Minority), (Race, Ethnicity), (Disparities, Determinants), and (Facilitators, Barriers). Searches were conducted in PubMed, Health Evidence, Public Health + , and the National Institute of Health and Care Excellence.

### Review criteria

We considered studies done in the USA as race is a social construct and the experience and impacts of individual and systemic racism differ geographically. We focused on results among Black/African American individuals given the research question and aim to identify specific determinants within this group; however, we did include studies with multiple racial groups represented. We focused on studies that included individuals aged 40–74 years to match the population eligible for average-risk breast cancer screening. Publications in the 3 years prior to evidence review were prioritized acknowledging determinants may change over time (e.g., with technology advancements such as online scheduling or policy changes allowing for mammogram scheduling without primary care provider (PCP) referral) and in keeping with methods in the National Collaborating Centre for Methods and Tools Rapid Review Guidebook [68].

### Critical appraisal and evidence synthesis

Critical appraisal was guided by the 6S Pyramid framework developed and made available by the National Collaborating Centre for Methods and Tools [69]. We categorized data by source (i.e., search engine), study type (e.g., single study, meta-analysis), population, and results.

### Interviews with mockup

#### Objective

Our objective was to elicit determinants to breast cancer screening among Black women living in western Washington as well as feedback about an initial mockup of the chatbot through interviews with community members.

### Interview guide

**Fig. 1:**
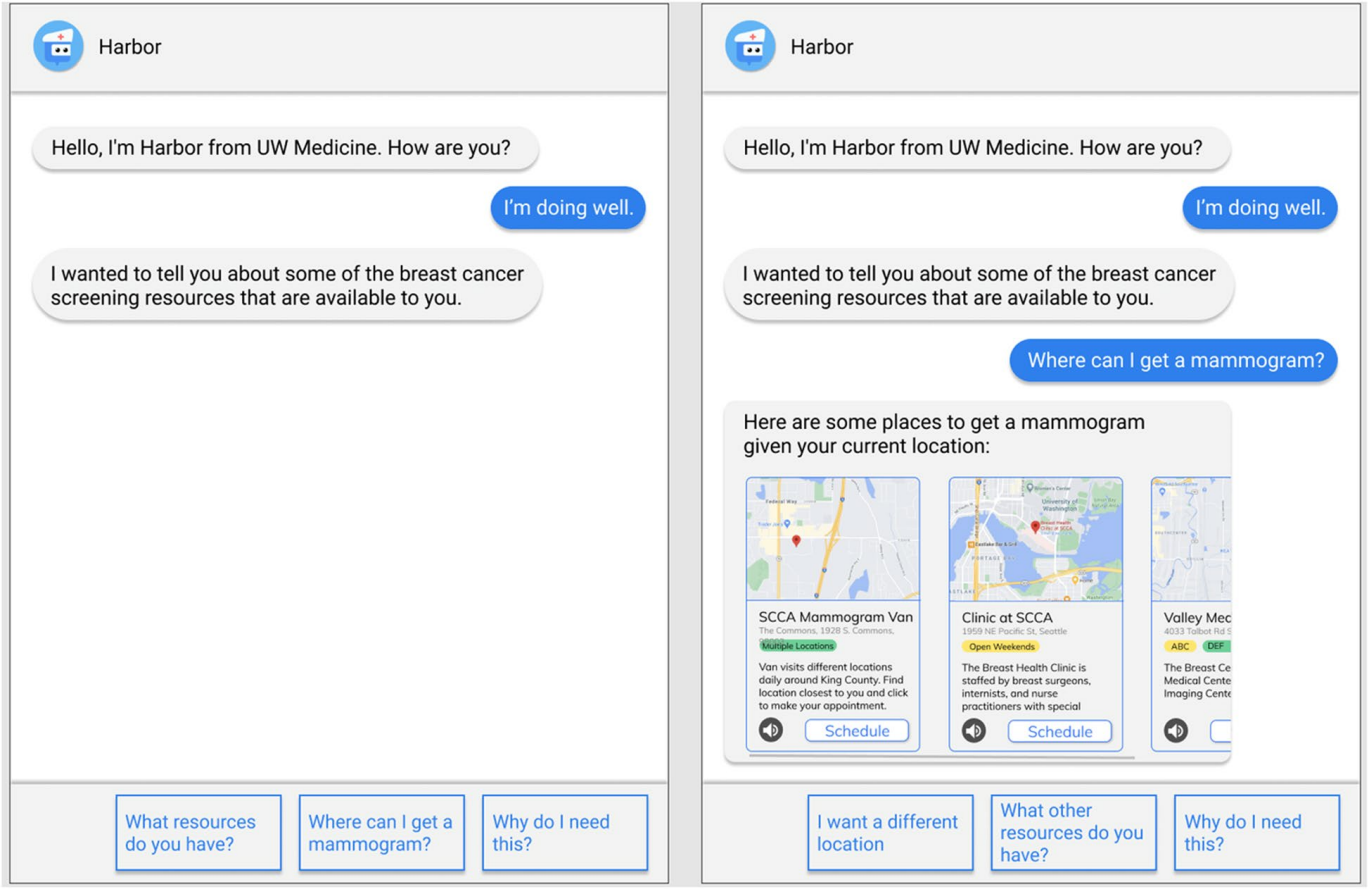
Initial mockup

The interview guide was developed by our research team with additional input from members of the Breast Health Equity committee—a health system committee including operational leaders, physicians, and researchers dedicated to addressing inequities in care related to breast cancer screening, diagnosis, and treatment (Additional file 1: Appendix A). While we incorporated feedback from committee members after the guide was drafted, we did not pilot test with community members before starting interviews. Questions focused on determinants to breast cancer screening and past experiences with breast cancer screening. Additionally, two members of the research team (G.H. and R.L.) created a mockup of the chatbot tool including several mockups of a chatbot for breast cancer screening outreach (Fig. 1).

### Sample and recruitment

We used convenience sampling through fliers posted in primary care clinics and email to the research team’s established community networks to identify and recruit individuals who identified as Black/African American women between the ages of 40 and 74 years and lived in either King or Pierce counties in Washington state. We recruited 21 individuals for interviews which we estimated would be adequate to provide sufficient data for understanding our research question [70].

### Procedure

Two members of the research team (B.H.H., A.G.) conducted interviews (*n* = 21); the community engagement lead on the team (B.H.H.) conducted the vast majority (*n* = 18). Interviews were conducted via Zoom videoconferencing technology and were audio recorded. In addition to questions regarding determinants to and experience of breast cancer screening, we showed participants screenshots of the initial mockup for the chatbot tool and asked specific questions for feedback. No field notes were collected during/after interviews. All interviews were transcribed.

### Analysis

Four members of the research team (R.L. and three undergraduate students listed in acknowledgements: A.A., N.S., X.S.) read and coded the transcripts to generate and refine themes through several iterations until consensus was reached. Each interview was analyzed and coded once by individuals on the research team using a directed content analysis approach; codes were then discussed as a team [5, 71]. Deductive codes were created using prior research organizing breast cancer screening barriers as personal, structural, and clinical [34]. Inductive codes emerged from a close reading of an initial subset of the transcripts and were added to the codebook (Additional file 1: Appendix Table B). Qualitative data analysis resulted in themes around the chatbot design, and determinants to breast cancer screening. We facilitated an ideation workshop with the research team and Breast Health Equity committee to brainstorm how this research might address the themes brought up in the interviews. We used a 2 × 2 prioritization matrix as a tool to identify the most impactful and feasible ideas that arose. This analysis was used to develop an early chatbot prototype. Results were shared with participants in a newsletter with invitation to respond to interpretation and/or presentation prior to manuscript submission (Additional file 1: Appendix C).

### Focus groups

#### Objective

The objectives were to elicit feedback on an early static prototype of the chatbot tool informed by the interviews.

#### Early prototype creation

The research team developed an early static prototype of the chatbot tool iterating on the initial mockup using themes and feedback that emerged from qualitative data analysis of the individual interviews (Fig. S1). In addition to the prototype screens, we included short videos with questions and answers to questions such as—“Why should I get screened?”, “What can I expect from a mammogram?”, “What happens if the mammogram is abnormal?”.

#### Focus group guide

The focus group guide was developed by our research team with additional input from members of the Breast Health Equity committee (Additional file 1: Appendix D). The guide included questions about perceptions of, engagement with, and usability of the chatbot based on the prototype screens and videos.

#### Sample and recruitment

The same sampling and recruiting methods were used for the focus groups as were used for the individual interviews. We conducted three focus groups with a total of nine participants.

### Procedure

Focus groups were led by B.H.H. and joined by multiple members of the research team (V.F., A.G., R.L., L.M.M.). We showed participants three example interactions with the chatbot prototype, (1) patient-initiated scheduling of a mammogram, (2) system-initiated patient education, and (3) system-initiated re-scheduling, and asked specific questions for feedback. The same procedures were followed as for the individual interviews; team members debriefed after each focus group.

#### Analysis

We used template analysis with pre-defined domains derived from focus group questions and interview themes to analyze focus group content [72]. Template analysis may be used as a rapid qualitative analysis approach for focus group data [73]. Template domains were agreed upon by investigators (L.M.M., G.H., R.L., B.H.H.). One investigator (L.M.M.) then reviewed focus groups and conducted content analysis using the templates. The completed templates were summarized in a matrix for data visualization and reviewed by all investigators; any disagreements were addressed and resolved. Results were shared with participants in a newsletter with invitation to respond to interpretation and/or presentation prior to manuscript submission (Additional file 1: Appendix C).

#### Synthesis: developing a causal pathway diagram

CPDs can help to map out the pathway of an implementation strategy as it is intended to work [6]. For innovative implementation strategies, CPDs can help establish and test theorized mechanisms. Conceptual frameworks help to inform pathway components and may be drawn from disciplines outside of implementation research for novel strategies.

From our rapid evidence review, qualitative interviews, and focus groups, we identified key determinants—both in the context of breast cancer screening and the chatbot implementation strategy—and hypothesized mechanisms. Our research team prioritized determinants that were identified across data sources (e.g., in interviews and in rapid evidence review). We focused CPD development on the initial engagement with the chatbot.

Using the selected key determinants, we worked to identify conceptual frameworks to develop a CPD. We expanded the search for conceptual frameworks outside of healthcare to include disciplines such as marketing and computer science that are relevant to the implementation strategy. We selected frameworks based on relevance to and connection of our implementation strategy and proposed mechanism. We used the conceptual frameworks to inform mechanisms through which we hypothesize the implementation strategy to work and moderators which could increase or decrease strategy effect via the strategy mechanism. We iterated on the CPD as a team and received feedback from the OPTICC center team (B.J.W. and A.R.L.).

## Results

### Informing CPD development: identifying and prioritizing key determinants

In the rapid evidence review, 41 relevant studies were identified out of 114 search results. A narrative synthesis was written summarizing determinants identified in the literature (Additional file 1: Appendix E). Determinants identified were cataloged and prioritized based on relevance to the implementation strategy. For example, one study found perceptions of lower quality of care if mammograms were done in a mobile clinic setting; we did not include this as a priority determinant because this would not be particularly modifiable in the chatbot design [33]. Priority determinants included facilitators such as having personal or family history of breast cancer and recommendations from PCPs and barriers such as medical mistrust (Table 1).

**Table 1:** Determinants to breast cancer screening from rapid evidence review and analysis of qualitative data

Breast cancer screening determinant*	Qualitative themes	Representative quotes
• **Lack of resources (cost, insurance, transportation)** [74, 75]	Participants discussed barriers including lack of resources, such as finances, transportation, work conflicts, anxiety about what to expect during the mammogram, and prior painful and/or negative experience	*“I was in West Seattle….a low-income area. And … there need to be more resources …that help out women of color… and explain what mammograms consist of. Talk about the cost of it. Talk about resources that individuals can tap into…to be able to get a mammogram.”—56 year old woman*
• Conflicts with work and/or other competing priorities		
• *Lack of PCP* [33, 76, 77]		
• **Anxiety about what to expect**		
• **Fear about pain, exposure to radiation or other negative outcomes** [33]** associated with procedure**		
• **Medical mistrust** [78]		
• **Prior negative experience including experiences of racism** [33, 77]		
• **Lack of knowledge about breast cancer screening** [33, 76, 78]		
• **Inadequate preparation/ information given prior to procedure** [33]		
• **Lack of discussion with friends and family** [76]		
• Tailored information about breast cancer	Participants emphasized the importance of outreach to get information about breast cancer screening to the community. It was mentioned that a barrier to screening is not being aware that it was something they should do	*“How do you know if you're carrying something around, you're sick and you're not knowing what it is, and when you get to the hospital, they diagnosed … you. But there's things that you could have done prior, if you was told. Some people don't know how to reach out.”—56 year old woman*
• **Family or personal history of breast cancer (or other cancers) as facilitator to screening** [33, 76, 79, 80]		
• **Recommendations from PCP**	Participants discussed lack of or equivocal recommendations by physicians for breast cancer screening, even if they initiated discussion. They also discussed reminders, the time it takes to make an appointment and time until appointment as determinants to scheduling screening	*“And now, I just need to bring it up again to my physician. Because I did talk to her about it two to three appointments ago, and she's not putting the referral.” – 42 year old woman*
• **Advocacy (or lack of) from PCP**		
• Time spent to make an appointment		
• Time until appointment		
• **Health-related social support** [33]		
		*“I don't think I have been screened this year because of the COVID-19. I've probably seen an email, which is kind of not really personable…because I think in the past I would've got a call…so it didn't make it as urgent or important at the top of the list.” – 52 year old woman*

^*^Plain text determinants emerged from interviews and/or focus group only, italicized determinants arose from rapid evidence review only, bolded determinants were present in both evidence review and interviews and/or focus groups

One priority barrier that emerged from the rapid evidence review was lack of knowledge about breast cancer screening; prior literature recommended patient education to explain and help individuals learn about the process of getting a mammogram [33, 76, 78]. This informed our design of the initial mockup and early chatbot prototype as a patient education and scheduling tool.

For the qualitative data analysis, we interviewed 21 of 39 individuals who responded to recruitment and completed a screening survey. Only 4 of 39 were ineligible due to living outside the 2 designated counties. Several people invited for interviews had to cancel due to schedule conflicts. All interviews were completed once started (no one dropped out of the study after starting an interview). Interview participants all identified as Black/African American, were between the ages of 40 and 69 years, and several were multilingual. We did not collect specific demographic characteristics for focus group participants. Focus group participants signed up for 1 of 4 focus groups; we did not have anyone drop out of focus groups once started.

In the qualitative analysis of interviews and focus groups, we elicited facilitators and barriers to breast cancer screening. Most of the determinants that emerged from interviews and focus groups were also identified in the evidence review (Table 1). Facilitators that appeared in evidence review and interviews and/or focus groups included recommendations and/or advocacy from a PCP, health-related social support, family or personal history of breast cancer, and adequate preparation before a mammogram (lack of preparation was framed as a barrier in evidence review). Overlapping barriers included lack of resources (e.g., cost, insurance, transportation), anxiety about what to expect, fear about negative outcomes associated with the procedure (e.g., pain), medical mistrust, prior negative experiences with the health system (including experiences of racism), lack of knowledge about breast cancer screening, lack of discussion with family and friends, and lack of clear recommendation from a PCP. Some determinants that arose from the interview and focus group data were not present in the evidence review but were prioritized given relevance to the implementation strategy. For example, participants identified the time spent to make an appointment and the time until the appointment as moderators to scheduling a mammogram (i.e., a barrier if time to make an appointment and time until appointment is long and facilitator if time to make an appointment and time until appointment is relatively short). In terms of initial reactions to the chatbot mockup, 18 out of 20 participants asked thought that the chatbot would be useful for scheduling (one participant was not asked this question).

In the template analysis of focus groups, we elicited facilitators and barriers to and feedback about the chatbot implementation strategy (Table 2; Additional file 1: Appendix F). Participants appreciated the purpose of the chatbot but thought that in many ways it fell short.

**Table 2:** Focus group themes

Domain	Theme(s)	Representative quotes
Motivation to screen	- Need to overcome competing priorities	*“I’m trying to feed my baby. I’m trying to get my kids clothes."(Participant, Focus Group 2)*
Reactions to media	- Positive reactions to videos	*“It's like, "Oh, that looks like me. Oh, that looks like somebody I can relate to."(Participant, Focus Group 1)*
	- Familiarity—liked seeing people who looked like them in image of a woman getting a mammogram	*“it's going to be important that whoever is involved in this not only looks the same skin color and ethnicity but age-wise, too, so that makes them more relatable, like someone who has actually had a mammogram themselves or who is old enough that needs one, I think would be important too.” (Participant, Focus Group 2)*
Reactions to content	- Appreciated discussion of cost	
	- Thought that question asking what prevented you from making your appointment sounded judgmental	
Perception of chatbot	- Some skepticism in multiple groups regarding chatbot persona	*“My first impression would be does she really know what she's talking about? Because just from the picture, I don't know. Yeah, that's what I think.” (Participant, Focus Group 3)*
		*“We don't just want her to just be a random name on the paper. She needs to represent what she's trying to teach us.” (Participant, Focus Group 2)*
Comfortability	- Majority of participants thought they would feel comfortable using chatbot although some skepticism about using artificial intelligence	*“got a problem with that whole Big Brother thing.” (Participant, Focus Group 3)*
Trust	- Expressed privacy concerns about chatbot	
Usefulness	- Saw chatbot as particularly useful for younger women/ first time screening	*“I'd just like to see more of our daughters and our daughter's friends, just to come together as a group and just have the knowledge, just so we get to tap in on that. You know, we know that they may not know, and their friends may not know. So just kind of, give more of an outlook on everything for them as well.” (Participant, Focus Group 1)*
Relatability	- Concern about cultural inclusiveness—felt that it wasn’t personalized outside of community partner involvement	*“And it just didn't speak to me as being a Black woman. That's what I'm going to say. But, you know, let's just be honest. Who made the app?” (Participant, Focus Group 2)*
Desired content	- More information about self-exams	*“I would like to have all of it, even the statistics because for me, I would want to go and encourage someone else to get a mammogram. And sometimes, not a lot of statistics, but just knowing among African Americans, that statistics, because a lot of us don't get mammograms because we've heard about the negative things instead of the positive things. So, yeah, I would want to know all of it.” (Participant, Focus Group 3)*
	- More breast cancer data about Black women specifically	
	- More information about how to prepare for mammogram	
		*“I used to believe that certain diseases were only for white people.” (Participant, Focus Group 2)*
		*“We got to come to the future, and feel comfortable in talking about our health, our breasts, all types of cancer. So some kind of way in there, explain the reason why women of color are disproportionate in this fight for cancer. Knowledge, communication, openness.” (Participant, Focus Group 1)*
Desired features/ functions	- Appointment reminders	
	- Include ways to make breast cancer screening social, e.g., “mammogram parties”	
Usability	- Should be efficient—able to schedule quicker than a phone call	*“And I can go right here and get it all done and be finished in 15, 20 min as opposed to being on the phone a half hour… I would definitely use it” (Participant, Focus Group 3)*
	- Did not want to download an app to use

“I mean because that's what the app is for… To kind of make us feel… to draw us in and make us feel taken care of and informed. Educated.” (Participant, Focus Group 2).

Participants expressed mistrust in the chatbot persona, questioning the chatbot’s credibility and describing privacy concerns and intent. They emphasized the importance of cultural inclusivity and familiarity but did not feel like the chatbot prototype achieved these goals.

“I do agree with the fact that it needs to be more culturally inclusive and appropriate for us. I didn't feel like it was personalized outside of [B.H.H.’s] involvement, there was nothing that really spoke to our people.” (Participant, Focus Group 2).

The chatbot presented to the focus groups was named “Sesi” which means “sister” in Sotho, a Bantu language spoken mostly in Southern Africa. Participants expressed frustration about conflating African and Black American experience.

“Sometimes, because we're Black, other communities patronize on us being Black… they just patronize us as if we know what it is to be in Africa and we don't. We've never been to Africa. We still have the same issues, yes, but we've never been there so we can't relate to certain things or cultures that have because we don't have that. We've never, that was not brought along with us here.” (Participant, Focus Group 3).

They questioned the value-add of the chatbot presumed to be an app that would require effort to download onto a phone but might only be used once a year. Though participants did think that they would use the chatbot if it could be used to schedule a mammogram more efficiently than by telephone conversation.

Overall, mistrust was a major theme in both qualitative data analyses and rapid evidence review. In interviews and focus groups, mistrust arose both in the context of interactions with the health care system and the chatbot technology. At the same time, participants noted aspects of the chatbot that increased trust and engagement. For example, they felt reassured to see women who looked like themselves in the chatbot interaction, such as an image of a Black female mammography technician. Given these findings, we decided to focus on optimizing trust in the design of the initial chatbot engagement.

### Causal pathway diagram

**Fig. 2:**
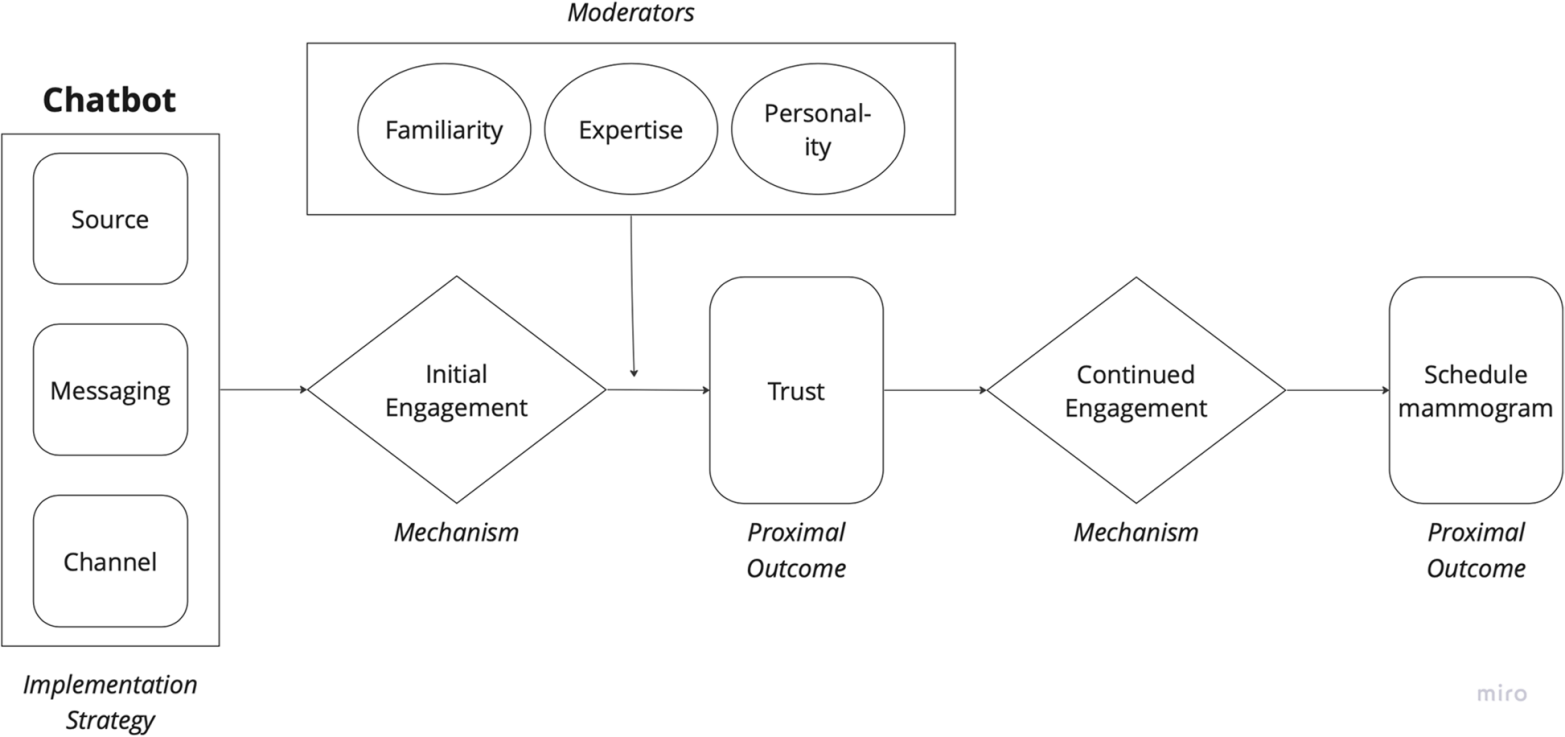
Causal pathway diagram

We drew from multiple theoretical frameworks regarding trust as a determinant to the evidence-based intervention (breast cancer screening) and the implementation strategy (chatbot) (Table 3). The persuasive health message framework for developing culturally specific messages details source, channel, and message as distinct components in health messaging and has been used in prior breast cancer screening campaigns [81, 82]. We defined our implementation strategy components using these conventions—source (i.e., chatbot persona—communication style and identity), channel (i.e., form of message delivery, e.g., SMS text), and message (i.e., content of messages) (Fig. 2). As we focused first on the initial engagement with the chatbot, we attended to source credibility to engender trust. We drew from a conceptual framework in marketing that identifies expertise, homophily, and trustworthiness as characteristics of source credibility—or the belief that a source of information can be trusted [83]. Finally, to capture trustworthiness in technology, we used a conceptual framework regarding trust in artificial intelligence which includes personality and ability as human characteristics that are important drivers of trust in AI [84].

**Table 3:** Integration of models/frameworks in the causal pathway diagram

Model/framework	Contribution
Persuasive Health Messaging Framework [82]	Informed core components of chatbot (source, messaging, channel)
Characteristics of source credibility on consumer behaviour [83]	Informed moderators homophily and expertise influencing trust
Trust in artificial intelligence, machine learning, and robotics [84]	Informed moderator personality as influencing trust

We used the CPD to model how we might address the barrier of mistrust using initial engagement with the chatbot as a mechanism and trust as a proximal outcome. Using the conceptual frameworks described above, we posited moderators to be (1) chatbot expertise, (2) chatbot designed for familiarity (i.e., homophily), and (3) chatbot personality or communication style. With our components defined, we created a CPD (Fig. 2).

## Discussion

We presented a case study example of the use of HCD methods to inform and build CPDs to design an implementation strategy rooted in causal perspective and informed by community partners. This approach addresses gaps in the use of implementation strategies including identifying and prioritizing determinants and knowledge of strategy mechanisms [6, 66].

Our work highlights the value of HCD methods which integrate end-users in the development of innovative implementation strategies and provides an approach for community/partner engagement that is especially useful when designing technology-based strategies. By using HCD methods, we were able to elicit determinants to both the evidence-based practice (breast cancer screening) and the proposed implementation strategy (chatbot). Moreover, we received nuanced feedback about the chatbot in its current design rather than as a hypothetical strategy (as visual tools in qualitative research can enhance the quality and clarity of data) [85]. By doing so, we gained important insights about the implementation strategy—e.g., our early prototype design lacked the depth of cultural inclusivity and familiarity needed to elicit trust and promote use of the chatbot despite being informed by breast cancer screening determinants elicited in our initial interviews. These insights were integral to the CPD development and prioritization of trust as a determinant to chatbot use and subsequent breast cancer screening.

Using HCD methods meant that we brought community partners into the design process in the earliest stages. Having text and visual content to react to allowed community partners to identify specifically what they liked and disliked about the design and how they would word chatbot messages differently—giving very concrete feedback to incorporate into future design iterations. There have been many calls to incorporate health equity in implementation science frameworks, strategies, and outcomes [65, 86–89]. Integrating the perspectives of populations with marginalized identities into the development of implementation strategies can help to address health equity more effectively and mitigate intervention-generated inequities [90, 91].

The CPD which was constructed by data from HCD methods directly informed our next steps in development of the chatbot prototype—(1) a factorial design experiment measuring degree of trust and engagement with different chatbot personas and (2) co-design sessions to craft chatbot messaging. While our case example details the design of an innovative implementation strategy, we believe this approach could be useful in tailoring a broad spectrum of implementation strategies and adds to existing literature on methods to tailor implementation strategies [92].

## Limitations

Our case study example has several limitations. Topically, while most people will have access to SMS-based interventions, this intervention will not be accessible and/or acceptable for all eligible patients [51]. During this work, we received feedback that the chatbot may be especially effective among younger age groups, but that uptake may be lower among older adults. As the USPSTF guidelines are expected to change to recommend earlier screening starting at 40 years, this implementation strategy could be particularly acceptable to outreach to newly eligible patients [93]. We readily acknowledge that a single intervention will not fully address breast cancer screening inequities and should be implemented as one part of a multi-faceted health system approach.

Methodologically, the chatbot development case example could have been strengthened using a determinant framework. We would encourage investigators interested in this approach to incorporate determinant frameworks in initial evidence review and data collection. Our methodological approach could also be strengthened by increasing community engagement [10, 14]. While we incorporated several elements of community-engaged research (including community members on the research team, having a trained community member conduct research with community participants, and community member co-authorship), we could have further expanded community participation (e.g., seek community input in initial intervention design, create a community advisory board).

## Conclusions

The use of interdisciplinary methods is core to implementation science [94]. HCD is a particularly synergistic discipline with multiple existing applications of HCD to implementation research. We present an extension of this work and an example of the potential value in an integrated approach of HCD and IS researchers and methods to combine strengths of both disciplines and develop human-centered, co-designed implementation strategies rooted in causal perspective and healthcare equity.

### Supplementary Information

**Additional file 1.** Appendices.

**Additional file 2: Fig. S1.** Early Chatbot Prototype.

## Data Availability

Aggregated qualitative data is available by request in accordance with a funding agreement. Disaggregated data is not available to maintain privacy and confidentiality of interview and focus group participants.

## Abbreviations

CPD: Causal pathway diagram
EHR: Electronic health record
EQUATOR: Enhancing the Quality and Transparency of health research
HCD: Human-centered design
PCP: Primary care provider
SMS: Short message service
TIDieR: Template for intervention description and replication
USPSTF: United States Preventive Services Task Force
