# Correction: Leveraging human-centered design and causal pathway diagramming toward enhanced specifcation and development of innovative implementation strategies: a case example of an outreach tool to address racial inequities in breast cancer screening

**DOI:** 10.1186/s43058-024-00586-9

**Published:** 2024-04-25

**Authors:** Leah M. Marcotte, Raina Langevin, Bridgette H. Hempstead, Anisha Ganguly, Aaron R. Lyon, Bryan J. Weiner, Nkem Akinsoto, Paula L. Houston, Victoria Fang, Gary Hsieh

**Affiliations:** 1 Department of Medicine, University of Washington School of Medicine, 908 Jeferson St, Seattle, WA 98104 USA; 2 Department of Human Centered Design and Engineering, University of Washington, Seattle, WA USA; 3 Cierra Sisters, Seattle, WA USA; 4 Department of Medicine, University of Texas Southwestern Medical Center, Dallas, TX USA; 5 Department of Psychiatry and Behavioral Sciences, University of Washington School of Medicine, Seattle, WA USA; 6 Departments of Global Health, University of Washington School of Medicine, Seattle, WA USA; 7 Health Systems and Population Health, University of Washington School of Medicine, Seattle, WA USA; 8 UW Medicine, enterprise health system of the University of Washington, Seattle, USA

**Correction** **:** **Implement Sci Commun 5, 31 (2024)**

**https://doi.org/10.1186/s43058-024-00569-w**

In the Original Article [1], the name of co-author Bridgette H. Hempstead was misspelled as Bridgette R. Hempstead. The author’s initial throughout the manuscript and in the Authors’ Contribution section, ‘B.R.H’ was also incorrect, as it should have been ‘B. H. H’.

This Correction article shows the correct spelling of the name. The original article has been corrected.
