# Supplementary material for: Leveraging human-centered design and causal pathway diagramming toward enhanced specification and development of innovative implementation strategies: a case example of an outreach tool to address racial inequities in breast cancer screening
Source: Implement Sci Commun. 2024 Mar 28;5:31. doi: 10.1186/s43058-024-00569-w (PMC10976783; doi:10.1186/s43058-024-00569-w)
Supplement: Supplementary file 1 — Additional file 1. Appendices. [file 43058_2024_569_MOESM1_ESM.pdf]

Appendix A. Interview Guide

Interview guide

1. What do you know about breast cancer screening (mammograms)?

2. Do you have a primary care provider?

    a. If yes, has your PCP talked to you about breast cancer screening? Has anyone else from your primary care clinic talked to you about breast cancer screening? How was that experience?

3. Who else have you discussed breast cancer screening with (other than health care professionals)?

4. Have you had breast cancer screening (mammogram)?

    a. If yes

        i. Why did you have breast cancer screening?

        ii. Where did you have breast cancer screening? (clinic, hospital, mobile van?)

        iii. Did anything make it easier or harder to get breast cancer screening?

    b. If no

        i. Why haven't you had breast cancer screening?

        ii. Have you ever been recommended to have breast cancer screening?

        iii. If you were to get breast cancer screening, where would you get it and when (what time, day of week)?

4. Have you had challenges with:

a. Scheduling appointments
b. Locating screening sites
c. Knowing whether and when you should get a mammogram
d. Other challenges?

5. Has the COVID-19 pandemic prevented you from getting screening or affected how you think about screening?

6. What else would you like to share that we haven't covered yet?

7. [Tool testing]

Our design team is working on an app to connect patients to more information about breast cancer screening. I'd like to show you a few screens to get your feedback.

Show them one screen at a time.

1. Intro screen, chatbot where users can ask some questions about screening.

+ What kinds of questions would you like to ask the chatbot?

2. Scheduling screen, one of the key functions would be to help patients find screening locations.

+ Would a feature like this be useful (it might tie in with 4b above). What other features would you like? How would these features work? (What questions are missing?)

3. Barriers screen, another key function would be to use the chatbot to address some common concerns patients may have.

+ What do you like about this app?

+ What do you not like about this app?

+ Would you use this app to ask these questions? When would you use this?

+ What would you change about this app, if anything?

Appendix B. Qualitative Analysis of Interviews: Code Descriptions

CODE TITLE	DESCRIPTION
ADVOCACY	Participant discusses importance of breast cancer screening advocacy and/or their efforts to advocate for screening.
ANNUAL SCREENING PRACTICES	Participant mentions whether or not they do an annual screening for breast cancer.
MISINFORMATION ONLINE	Participant mentions facing misinformation about breast cancer screening on the internet.
PERCEPTIONS OF SCREENING	Participant mentions their perception of breast cancer screening and reflects on their experience.
STRUCTURAL SUPPORT	Participant mentions how healthcare provider supports them with scheduling and other logistics to address barriers to breast cancer screening.
SYSTEMIC RACISM IN HEALTHCARE	Participant mentions systemic racism in healthcare, such as inequities in accessing screening.
QUOTES	Participant mentions an interesting quote.
**BARRIER**	Participant mentions barriers to breast cancer screening.
COVID-19 IMPACT	Participant mentioned that they have challenges in accessing screening due to COVID-19 as an unexpected circumstance.
**CLINICAL BARRIERS**	Clinical barriers to breast cancer screening care including lack of patient education or information.
COMMUNICATION BARRIERS	Participant mentions that they had a lack of information about breast cancer screening, or faced barrier in understanding information.
LACK OF INFORMATION FROM PROVIDER	Participant mentions that their healthcare provider did not discuss breast cancer screening with them.
LACK OF OUTREACH	Participant mentions lack of outreach from healthcare providers regarding breast cancer screening.
UNFAMILIAR WITH BREAST CANCER SCREENING	Participant mentions a time when they were unfamiliar with breast cancer screening.
EXAM PROCESS	Participant discusses pain or discomfort with the mammogram exam process.
**PERSONAL BARRIERS**	Personal barriers to care including lack of trust or negative healthcare experiences.
PAINFUL EXPERIENCE	Participant has experienced or heard about a painful breast cancer screening process.

PROCRASTINATION	Participant mentions procrastination in scheduling breast cancer screening, for example due to painful experience.
**STRUCTURAL BARRIERS**	Structual barriers to care, such as lack of insurance, facilities, providers, or transportation.
COVID-19 AFFECTING HEALTHCARE SYSTEM	Participant mentions COVID-19 as a barrier to scheduling a screening, for example appointment cancellations by the hospital.
LACK OF ADVOCACY	Participant mentions lack of advocacy from healthcare providers as a barrier, such as not encouraging them to get screened.
LACK OF INSURANCE	Participant mentions lack of healthcare insurance as a barrier.
**CHATBOT**	Participant mentions feedback related to the chatbot.
ADDITIONAL CHATBOT FEATURES	Participant mentions additional features that they would like to do through the chatbot interface.
ADDITIONAL CHATBOT QUESTIONS	Participant mentions additional questions that they would like to have answered through the chatbot interface.
CHATBOT PAIN POINTS	Participant mentions a pain point or difficulty they would have with the chatbot interface.
CHATBOT USE IS INEFFICIENT	Participant mentions that using the chatbot interface for asking questions about breast cancer screening is inefficient.
CHATBOT USE CASES/SCENARIOS	Participant describes a use case detailing when and how they would use the chatbot interface.
SUCCESSES WITH THE CHATBOT	Participant mentions what they liked and/or what worked well about the chatbot interface.
**INTERVENTION TO ADDRESS BARRIERS**	Participant suggests an intervention or approach to help with addressing barriers to breast cancer screening (e.g. clinical, personal, structural)
INFORMATION INTERVENTION	Intervention to spread awareness or share information about breast cancer/breast cancer screening.
PROCESS INTERVENTION	Intervention to directly improve the breast cancer screening process.
TECHNOLOGICAL INTERVENTION	Intervention that uses technology to address barriers to breast cancer screening, outside of the chatbot interface.
**PREVIOUS KNOWLEDGE OF BREAST CANCER SCREENING**	Participant discusses previous knowledge about breast cancer/breast cancer screening.
AWARENESS	Participant discusses personal awareness about breast cancer/breast cancer screening.
FAMILY HISTORY	Participant discusses family history of breast cancer.
LACK OF PREVIOUS KNOWLEDGE	Participant mentions lack of previous knowledge about breast cancer/breast cancer screening.
PROVIDER DISCUSSES SCREENING	Participant mentions conversations they have had with their healthcare provider about breast cancer screening.

Appendix C. Newsletter shared with participants

Update in project to address inequities in breast cancer screening

April 7, 2023

Project Team
Leah Marcotte
Raina Langevin
Bridgette Hempstead
Anisha Ganguly
Aaron Lyon
Bryan Weiner
Nkem Akinsoto
Paula Houston
Victoria Fang
Gary Hsieh

Thank you so much for helping us with the project to design a chatbot to encourage and inform Black/African American women about breast cancer screening. A chatbot is a tool like iPhone Siri or Amazon Alexa that you can have a conversation with. We showed everyone who we interviewed or who were in focus groups a version of the chatbot. We have used your feedback to continue to improve the chatbot design. We are submitting an article that discusses our approach in getting feedback from interviews and focus groups and how that impacted the design.

In this newsletter, we want to walk you through this approach as we do in the article and share some of the quotes from the interviews and focus groups that informed this work. If you see a quote that you said, please make sure you feel like it is used in the way that you intended. If you have any concerns, we can edit or delete the quote. (Please email Bridgette Hempstead at cierra_sisters@hotmail.com if you would like to edit or delete a quote or if you'd like to see the full article we are submitting.)

What makes it easier or harder to get breast cancer screening?

We learned about reasons why women are not able to get mammograms:<br>- Cost<br>- Transportation<br>- Work conflicts<br>- Anxiety about what to expect during the mammogram<br>- Prior painful experience<br>- Prior negative experience	*"I was in West Seattle….a low-income area. And … there need to be more resources …that help out women of color... and explain what mammograms consist of. Talk about the cost of it. Talk about resources that individuals can tap into…to be able to get a mammogram." - 56 year old woman*<br><br>*"I'm trying to feed my baby. I'm trying to get my kids clothes."(Participant, focus group 2)*
We heard about the importance of getting information about breast cancer screening to the community. One reason why people don't get screened is because they are not aware they should.	*"How do you know if you're carrying something around, you're sick and you're not knowing what it is, and when you get to the hospital, they diagnosed … you. But there's things that you could have done prior, if you was told. Some people don't know how to reach out." - 56 year old woman*
We heard that sometimes primary care physicians are not giving clear recommendations about breast cancer screening.<br><br>We heard about the importance of reminders.<br><br>We also heard about the time it takes to schedule a mammogram as being a barrier to screening.	*"And now, I just need to bring it up again to my physician. Because I did talk to her about it two to three appointments ago, and she's not putting the referral." – 42 year old woman*<br><br>*"I don't think I have been screened this year because of the COVID-19. I've probably seen an email, which is kind of not really personable…because I think in the past I would've got a call…so it didn't make it as urgent or important at the top of the list." – 52 year old woman*

What were reactions to the chatbot design?

Chatbot design showed to focus groups:

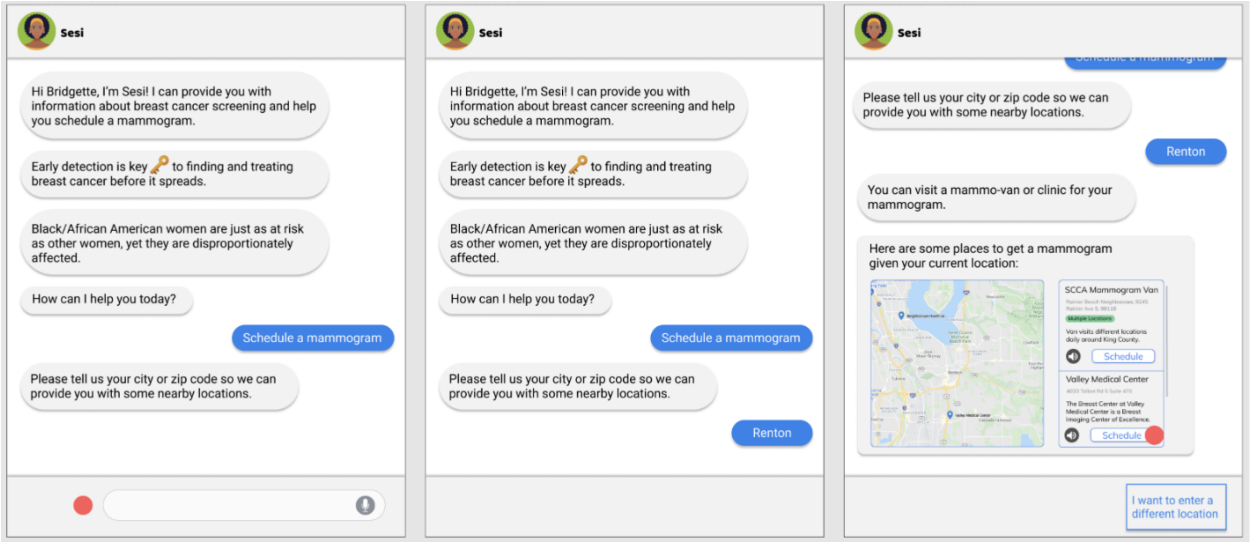

- Generally liked the videos (focus groups)<br>- Liked seeing people who looked like them in image of a woman getting a mammogram (interviews)	*"It's like, "Oh, that looks like me. Oh, that looks like somebody I can relate to."(Participant, Focus Group 1)*<br><br>*"it's going to be important that whoever is involved in this not only looks the same skin color and ethnicity but age-wise, too, so that makes them more relatable, like someone who has actually had a mammogram themselves or who is old enough that needs one, I think would be important too." (Participant, Focus Group 2)*
- Appreciated discussion of cost<br>- Thought that question asking what prevented you from making your appointment sounded judgmental	
- Skepticism in focus groups about the chatbot image and personality	*"My first impression would be does she really know what she's talking about? Because just from the picture, I don't know. Yeah, that's what I think." (Participant, Focus Group 3)*<br><br>*"We don't just want her to just be a random name on the paper. She needs to represent what she's trying to teach us." (Participant, Focus Group 2)*
- Skepticism about artificial intelligence<br>- Concerns about privacy and technology	*"got a problem with that whole Big Brother thing." (Participant, Focus Group 3)*
- Saw chatbot as particularly useful for younger women/ first time screening	*"I'd just like to see more of our daughters and our daughter's friends, just to come together as a group and just have the knowledge, just so we get to tap in on that. You know, we know that they may not know, and their friends may not know. So just kind of, give more of an outlook on everything for them as well." (Participant, Focus Group 1)*
- Concern about cultural inclusiveness – felt that it wasn't personalized outside of community partner involvement	*"And it just didn't speak to me as being a Black woman. That's what I'm going to say. But, you know, let's just be honest. Who made the app?" (Participant, Focus Group 2)*
- More information about self-exams<br>- More breast cancer data about Black women specifically	*"I would like to have all of it, even the statistics because for me, I would want to go and encourage someone else to get a mammogram. And sometimes, not a lot of statistics, but just knowing among African Americans, that statistics, because a lot of us don't get mammograms because we've heard*

- More information about how to prepare for mammogram	*about the negative things instead of the positive things. So, yeah, I would want to know all of it." (Participant, Focus Group 3)*<br><br>*"I used to believe that certain diseases were only for white people." (Participant, Focus Group 2)*<br><br>*"We got to come to the future, and feel comfortable in talking about our health, our breasts, all types of cancer. So some kind of way in there, explain the reason why women of color are disproportionate in this fight for cancer. Knowledge, communication, openness." (Participant, Focus Group 1)*
- Appointment reminders<br>- Include ways to make BCS social, for example, "mammogram parties"	
- Should be efficient – able to schedule quicker than a phone call<br>- Did not want to download an app to use	*"And I can go right here and get it all done and be finished in 15, 20 minutes as opposed to being on the phone a half hour... I would definitely use it" (Participant, Focus Group 3)*

Summary and Next Steps

Overall, we learned about what people found helpful about the chatbot and ways that it fell short.

- *"I mean because that's what the app is for… To kind of make us feel... to draw us in and make us feel taken care of and informed. Educated." (Participant, Focus Group 2)*

- *"I do agree with the fact that it needs to be more culturally inclusive and appropriate for us. I didn't feel like it was personalized outside of [Bridgette Hempstead's] involvement, there was nothing that really spoke to our people." (Participant, Focus Group 2)*

- *"Sometimes, because we're Black, other communities patronize on us being Black... they just patronize us as if we know what it is to be in Africa and we don't. We've never been to Africa. We still have the same issues, yes, but we've never been there so we can't relate to certain things or cultures that have because we don't have that. We've never, that was not brought along with us here." (Participant, Focus Group 3)*

We are continuing this work by getting feedback through surveys on different designs of the chatbot (example below). In the next few months, we will organize "co-design" sessions that include our research team and community members to help with the final design and messages for the chatbot. The next step will be to build the technology and send the chatbot through text message to those due for breast cancer screening.

We thank you so much for contributing to this work and welcome any additional thoughts and feedback.

Appendix D. Focus Group Guide

Focus Group Outline: *semi-structured interview format*

In these screens, the patient receives a message from Sesi that they are due for a mammogram. The patient asks for more information and Sesi guides them through questions about what to expect, etc. These screens include question & answer videos.

Motivation

Show the first screen:

1. Does this encourage you to schedule a mammogram?
2. Does the conversation feel engaging? If not, what additions would make you interested in continuing to participate with the app?
3. Is the sentence about self-exams confusing? Does it help you understand the difference between self-exams and mammograms?

Progress to the screen with the mammogram machine:

4. Does seeing the image of the mammogram machine make you more or less comfortable with proceeding?

After showing the last screen, go back to the first screen with Sesi logo:

Perception of Sesi

1. Would you feel comfortable to talk with Sesi about issues related to breast cancer screening?
2. How much do you trust Sesi?
3. Do you think you will use some of the information provided by Sesi?
4. How relatable was Sesi? What was your first impression of Sesi?

In these screens, the patient is opening the app and is using it to schedule a mammogram appointment.

After showing all of the screens:

Features/Functions

5. Would you prefer to have an option in the app to request a female mammography technician?
6. Are there other types of information that you would like from this chatbot?
7. What else would you like to use this chatbot for?

Usability

1. Based on the interactions that were shown, do you think that the system is easy to use?
2. Do you foresee any difficulties while interacting with Sesi?
3. Was there anything that Sesi said that was confusing?

In these screens, a patient previously scheduled an appointment, but they could not make their appointment. Sesi sends them a message to ask why they couldn’t make an appointment and helps them to reschedule.

Show the first screen:

Motivation

1. Would this encourage you to reschedule your appointment?

Appendix E. Rapid Evidence Review: Data Extraction Table

Title	Author (Year)	Type of publication (number of studies)	Setting	Population	Methodology	Result(s)	Result(s) among subgroups
1. Delayed or failure to follow-up abnormal breast cancer screening mammograms in primary care: a systematic review.	Reece (2021)	Systematic review (18 studies)	17 studies in United States and 1 in Netherlands	Women with inadequate abnormal mammogram follow-up of breast cancer screening mammograms	A systematic literature review to determine the extent of inadequate follow-up of abnormal screening mammograms in primary care and identify factors impacting on follow-up was conducted. Relevant studies published between 1 January, 1990 and 29 October, 2020 were identified by searching MEDLINE®, Embase, CINAHL® and Cochrane Library, including reference and citation checking. Joanna Briggs Institute Critical Appraisal Checklists were used to assess the risk of bias of included studies according to study design.	Factors influencing follow-up included physician-patient miscommunication, information overload created by automated alerts, the absence of adequate retrieval systems to access patient's results and a lack of coordination of patient records. Logistical barriers to follow-up included inconvenient clinic hours and inconsistent primary care providers. Patient navigation and case management with increased patient education and counselling by physicians was demonstrated to improve follow-up.	Women of ethnic minority and lower education attainment were more likely to have inadequate follow-up.
2. Breast cancer screening adherence rates and barriers of implementation in ethnic, cultural and	Ferreira (2021)	Systematic review (19 studies)	Global (Multiple countries)	Minority groups	The aim of the present study was to collect information from different minority groups worldwide, assess adherence to breast cancer screening and evaluate barriers or	Evidence was found that BC screening adherence among women that belong to minority groups depends on several	For African-American women, low educational level, younger age, medical mistrust, lack of knowledge

religious minorities: A systematic review.					limitations causing non-adherence, which should facilitate the development of effective interventions. A search was conducted through PubMed and Web of Science. Studies were considered as eligible if they met the following criteria: i) Female patients; ii) breast cancer screening program implemented in the country; iii) minority groups; iv) asymptomatic; v) report written in Portuguese or English; vi) study published from 2015 onwards.	factors, such as sociodemographic (high income, educational level and age); personal (fear, distrust of health professionals or healthcare system, lack of knowledge and pain or discomfort); ethical, cultural and religious (religious beliefs, role of men, family and community) and external (access to healthcare, sex of the physician and efforts to bring healthcare facilities closer to women).	and time, not having PCP are reasons for low adherence to breast cancer screening
3. Call to action: breast cancer screening recommendations for Black women	Oppong (2021)	Systematic review (5 screening guidelines)	United States	African American/Black women	We review breast cancer screening guidelines and address the lack of inclusion of the specific needs of Black women.	In order to equitably care for the health needs of Black women, high-risk designation would improve access to earlier screening and supplemental imaging including breast MRI.	
4. Breast Cancer Screening and Care Among Black Sexual Minority Women: A Scoping Review of the Literature from 1990 to 2017	Malone (2019)	Systematic review (15 studies)	United States	Black sexual minority women	This scoping review examines the literature from 1990 to 2017 of the breast cancer care continuum among Black sexual minority women, including behavioral risk factors, screening, treatment, and survivorship. A total of 91 articles were identified through PubMed, PsycINFO, and CINAHL	The 15 articles were primarily within urban contexts, and defined sexual minorities as lesbian or bisexual women. Across all the studies, Black sexual minority women were highly under-represented, and key conclusions are not	

					(Cumulative Index to Nursing and Allied Health Literature) databases.	fully applicable to Black sexual minority women. Sexual minority women had a higher prevalence of breast cancer risk factors (i.e., nulliparity, fewer mammograms, higher alcohol intake, and lower oral contraceptive use). Furthermore, some studies noted homophobia from health providers as potential barriers to engagement in care for sexual minority women.	
5. A Systematic Review of Barriers and Facilitators to Mammography in American Indian/Alaska Native Women	Jerome-D'Emilia (2019)	Systematic review (18 studies)	United States	American Indian and Alaska Native (AI/AN) women	A systematic search of MEDLINE and CINAHL databases identified relevant research studies published from 2007 to 2017.	Consistent with other low-income populations, socioeconomic factors were related to lower rates of screening in AI/AN women. However, some factors, such as reliance on the Indian Health Service, cultural issues, and traditionality were unique to this population. Factors that would facilitate screening for AI/AN women included a physician's recommendation, specifically if the	This systematic review revealed that there were very few intervention studies focused on breast cancer screening among AI women. The three intervention studies included were geographically dispersed with sites in Texas, North Carolina, and Washington. One study included posttests, which indicated an increased

						physician made a referral for a certain facility, or if the woman could be screened the same day she received the recommendation. Indeed such factors as having a higher income, more education, a usual source of care and insurance, and having had a recent physician's visit have been found to be facilitators in most if not all races and ethnicities of women, including AI/AN women. Consistent with other low-income populations, socioeconomic factors such as lack of insurance, lower income and less education, inadequate access to care, and the absence of a usual source of care were related to lower rates of screening in the AI/AN population.	understanding of the need for early detection and that either rising age or being childless can increase the risk of breast cancer. Doorenbos et al. (2011) recruited patients treated at a multidisciplinary community-based clinic, which treats individuals from more than 200 tribes and other low-income residents in the Pacific Northwest. This intervention was an attempt to evaluate the effect of targeted health messaging sent to individual patients in the form of a calendar. A control group received a calendar without preventive health messaging. Researchers then followed up via medical record review for 15 months. This study had a sample of 5,633 subjects, including a control group of 2,668

							(708 women aged 40 years and older) and an experimental group of 2,695 (722 women aged 40 years old and older). The calendar with health messages did not result in increased receipt of any cancer-related prevention outcomes compared with the calendar without health messages. For this study, Katz et al. (2008) used a case control design that involved an educational program provided by a lay health advisor for the cases and a brochure and physician letter sent to the controls. The sample was a random selection of women who received services through this rural provider and who had not been screened in the

							past year. Of the total sample of 851 women, 323 self-identified as AI. Women in the lay health advisor group compared with the women in the comparison group (i.e., those who received a physician letter and brochure about cervical cancer screening) displayed better belief scores and reduced barriers to mammography at follow-up.
6. Mammographic screening attendance among immigrant and minority women: a systematic review and meta-analysis	Bhargava (2018)	Systematic review (19 studies)	Europe, North America, and Oceania	Immigrant and minority women	A literature search of PubMed, Embase, Google Scholar, and Cochrane identified 1369 papers published between January 1995 and March 2016. In the review, we included 33 studies investigating mammographic screening attendance among immigrant and/or minority women. In a meta-analysis, we included 19 of the studies that compared attendance among immigrant and/or minority women with that among other women, using a random effects model.	Attendance was generally lower among immigrant and minority women compared to other women (46.2% vs. 55.0%; odds ratio = 0.64, 95% confidence interval = 0.56-0.73; $P < 0.05$, I2 = 99.9%). Non-Western immigrants had lower attendance rates than other immigrants.	

7. A systematic review of health promotion interventions to increase breast cancer screening uptake: From the last 12 years	Agide (2018)	Systematic Review (22 studies)	Global (Multiple countries)	Women	Online databases (PubMed/MEDLINE/PubMed Central, Ovid/MEDILINE, EMBASE, Web of Science and Google Scholar) were searched for studies published between January 2005 and January 2017. A quality coding system was assessed using Cochrane checklists for randomized controlled trial (RCT) and Downs and Black checklists for non-RCT. The score was rated for the included articles by each researcher independently and the average score is given accordingly.	Thirteen studies (59.10%) were conducted in the United States, 4 in Iran (18.18%), 2 in India (9.09%) and 1 each in Turkey, Saudi Arabia and Israel. The interventions were classified as 'individual-based', 'community-based', 'group-based teachings and training' and 'behavioral model based'. The majority of the studies showed favorable outcomes after health promotion interventions, including improvements in women's view of breast screening, breast self-examination and knowledge of breast screening.	
8. Effectiveness of patient-targeted interventions to increase cancer screening participation in rural areas: A systematic review	Rodriguez-Gomez (2020)	Systematic review (20 studies)	Global	Rural populations	An exhaustive literature search was performed in the most relevant bibliographic databases for biomedical research. The systematic review was reported according to the PRISMA guidelines.	Twenty studies assessing 37 interventions were identified. Most of the studies were conducted in the United States and targeted women. Ninety-seven percent of the interventions were aimed at increasing community	

						demand, 65% community access and 11% provider delivery. Our findings suggest that 21 of the 37 interventions using a multicomponent approach were effective in increasing breast, cervical and colorectal cancer screening in rural areas.	
9. Effectiveness of Interventions for Breast Cancer Screening in African American Women: A Meta-Analysis	Copeland (2018)	Meta-Analysis (14 studies)	United States	African American women	Articles published in English and in the United States, between January 1997 and March 2017, were eligible for inclusion if they (1) conducted psychosocial, behavioral, or educational interventions designed to increase screening mammography rates in predominantly African American women of all ages; (2) utilized a randomized, controlled trial (RCT) design; and (3) reported quantitative screening rates following the intervention.	Findings indicated that screening interventions for African American women were significantly more likely to result in mammography than control (OR = 1.56 [95 percent CI = 1.27-1.93], $p < .0001$). Although no patient or study characteristics significantly moderated screening efficacy, the most effective interventions were those specifically tailored to meet the perceived risk of African American women. Screening interventions are at least minimally effective for promoting mammography among African American	

						women, but research in this area is limited to a small number of studies.	
10. Identifying Equitable Screening Mammography Strategies for Black Women in the United States Using Simulation Modeling	Chapman (2021)	Primary research	United States	A 1980 U.S. birth cohort of Black and White women	An established model from the Cancer Intervention and Surveillance Modeling Network simulated screening outcomes using race-specific inputs for subtype distribution; breast density; mammography performance; age-, stage-, and subtype-specific treatment effects; and non-breast cancer mortality.	Biennial screening from ages 45 to 74 years was most efficient for Black women, whereas biennial screening from ages 40 to 74 years was most equitable. Initiating screening 10 years earlier in Black versus White women reduced Black-White mortality disparities by 57% with similar LYG per mammogram for both populations.	
11. Factors Associated with Breast Cancer Screening Adherence among Church-Going African American Women	Agrawal (2021)	Primary research	United States	919 African American, church-going women from Houston, Texas	Logistic regression analyses measured associations between breast cancer screening adherence over the preceding 12 months (adherent or non-adherent) and predisposing (i.e., age, education, and partner status), enabling (i.e., health insurance status, annual household income, employment status, patient-provider communication, and social support), and need (i.e., personal diagnosis of cancer, family history of cancer, and risk perception) factors, separately and conjointly.	Older age (predisposing: OR = 1.015 (1.007-1.023)), having health insurance and ideal patient-provider communication (enabling: OR = 2.388 (1.597-3.570) and OR = 1.485 (1.080-2.041)), and having a personal diagnosis of cancer (need: OR = 2.244 (1.058-4.758)) were each associated with greater odds of screening adherence. Overall, results suggest that	

						interventions which are designed to improve mammography screening rates amongst African American women might focus on broadening health insurance coverage and working to improve patient-provider communication.	
12. Mammogram Adherence Among Filipino American Women	Oviedo (2021)	Primary research	United States	Filipino American women	Logistic regression models determined the influence of predisposing, enabling and need variables with mammogram adherence.	Among all variables- breast cancer literacy, mammogram reminder, sociocultural deterrents, cultural beliefs, and years of residence in the United States-only a mammogram reminder from a healthcare provider was significantly associated with mammogram adherence.	
13. Breast Cancer Educational Needs and Concerns of African American Women Below Screening Age	Huq (2021)	Primary research	United States	African American women, family and community members	Data were collected through 30 key informant interviews with young African American women breast cancer survivors (diagnosed between 18 and 45), family members of African American women diagnosed between 18-45 years, and community organization leaders and healthcare providers who	Although most Breast Cancer Knowledge and Perceived Risk educational needs were consistent with those of older women, there were specific needs involving Cultural Reluctance in Health Disclosures	

					work with young African American women impacted by breast cancer. Data were coded and analyzed by multiple team members using template analysis.	and Breast Cancer Risk Reduction.	
14. Geographic Disparities in Late-Stage Breast Cancer Diagnosis Rates and Their Persistence Over Time	Mobley (2021)	Primary research	United States	Women	We examined all primary breast cancers diagnosed among all counties in 43 U.S. states with available data. We used spatial cluster analysis to identify hot spots (i.e., spatial clusters with above average late-stage diagnosis rates among counties). Demographic and socioeconomic characteristics were compared between persistent hot spots and those counties that were never hot spots.	Of the 2,599 counties examined in 43 states, 219 were identified as persistent hot spots. Counties with persistent hot spots (compared with counties that were never hot spots) were located in more deprived areas with worse housing characteristics, lower socioeconomic status, lower levels of health insurance, worse access to mammography, more isolated American Indian/Alaska Native, Black, or Hispanic neighborhoods, and larger income disparity. In addition, persistent hot spots were significantly more likely to be observed among poor, rural, African American, or Hispanic communities, but not among poor, rural, White communities. This analysis includes	

						a broader range of socioeconomic conditions than those included in previous literature.	
15. Changes in Mammography Use by Women's Characteristics During the First 5 Months of the COVID-19 Pandemic	Sprague (2021)	Primary research	United States	Women	We collected data on 461 083 screening mammograms and 112 207 diagnostic mammograms conducted during January 2019 through July 2020 at 62 radiology facilities in the Breast Cancer Surveillance Consortium. We compared monthly screening and diagnostic mammography volumes before and during the pandemic stratified by age, race and ethnicity, breast density, and family history of breast cancer.	Monthly screening mammography volume in July 2020 for Black, White, Hispanic, and Asian women reached 96.7% (95% CI = 88.1% to 106.1%), 92.9% (95% CI = 82.9% to 104.0%), 72.7% (95% CI = 56.5% to 93.6%), and 51.3% (95% CI = 39.7% to 66.2%) of the July 2019 pre-pandemic volume, respectively. Despite a strong overall rebound in mammography volume by July 2020, the rebound lagged among Asian and Hispanic women, and a substantial cumulative deficit in missed mammograms accumulated, which may have important health consequences.	
16. Project ScanVan: Mobile mammography services to decrease socioeconomic	Tsapatsaris (2021)	Primary research	NYC	Women	This is a retrospective cohort study, with permission to use de-identified data received from Project Renewal's ScanVan in 2019. Project Renewal ScanVan provides	In 2019, 66% (2499 of 3745) of patients who used the ScanVan were Hispanic & African American. 43% (1627 of 3745) of	Barriers to breast cancer screening include lack of medical insurance, limited access to care, and the

barriers and racial disparities among medically underserved women in NYC					clinical breast exams, mammograms, and health education to low-income women. The screening mammograms were conducted in the ScanVan and read by a board-certified radiologist. The electronic medical records were reviewed with respect to the patients' BI-RADS category, insurance status, race, and age. Descriptive statistics were performed and cancer detection rate, recall rate, and positive predictive values (PPV1, PPV2, PPV3) were calculated.	the women were uninsured, 15% (579 of 3745) of the women had Medicare, and 18% (676 of 3745) classified as other. 17 out of 3745 patients screened received a new diagnosis of breast cancer, corresponding to a cancer detection rate of 4.5/1000 screened. 258 were recalled, corresponding with a recall rate of 7%. The PPV1 was 6.5% (17/258); PPV2 was 29.8% (17/57); and PPV3 was 34% (17/50).	absence of a primary care physician. The mobile mammography screening van successfully overcame such barriers, providing uninsured women from racial minority groups with vital breast cancer screening and follow up care.
17. Evaluation of Patterns in Access to Breast Cancer Care and Breast Cancer Presentation in a Safety Net Patient Population	Choe (2021)	Primary research	United States	Women	We performed a retrospective analysis of patients with newly diagnosed breast cancer from 2014 to 2016 to evaluate how patients presented and accessed cancer care services in our urban safety net hospital. Patient demographics, cancer stage, history of breast cancer screening, and process of referral to cancer care were collected and analyzed.	Of the 202 patients identified, 61 (30%) patients were younger than the age of 50 and 75 (63%) were of racial minority background. Only 39% of patients with a new breast cancer were diagnosed on screening mammogram. Women younger than the age of 50 ($P < .001$) and minority women ($P < .001$) were significantly less likely to have had any prior screening	

						mammograms. Furthermore, in patients who met the screening guideline age, more than half did not have prior screening mammograms.	
18. Beyond Black and White: race and sexual identity as contributors to healthcare system distrust after breast cancer screening among US women	Dean (2021)	Primary research	United States	Black or White women	This cross-sectional study used intersectionality decomposition methods to assess the degree to which racial and SM identity contributed to disparate responses to the validated 9-item HCSD Scale. The sample included online survey participants identifying as a Black or White woman living in the US, with a self-reported abnormal breast cancer screening result in the past 24 months and/or breast cancer diagnosis since 2011.	Sexual minority (SM) identity emerged as the largest driver of Health care system distrust (HCSD) disparities; however, the combined racial and SM disparity persisted. Excluding sexual identity in HCSD studies may miss an important contributor. Interventions designed to increase the HCS's trustworthiness at the provider and system levels should address both racism and homophobia.	
19. Mammography Screening Among Latinas: Does Gender and Ethnic Patient-Physician Concordance Matter?	Mendoza-Grey (2021)	Primary research	NYC	Dominican women	We conducted structured interviews, in Spanish, with 419 Dominican women aged 40 years or older living in New York City. Using bivariate analysis and logistic regressions, we tested whether patient-provider gender, ethnic, and language concordance was associated with recent mammography when controlling for	Gender concordance predicted recent mammography after controlling for covariates ($\beta = 0.13$). Neither ethnic nor language concordance significantly predicted recent mammography. Our findings suggest that promotion of patient-provider	

					demographic covariates, breast cancer screening knowledge, and self-rated health.	gender concordance may help reduce health disparities among Latinos/as and other minority groups across the United States.	
20. Examining the Family Support Role of Older Hispanics, African Americans, and Non-Hispanic Whites and Their Breast Cancer Screening Behaviors	Cadet (2021)	Primary research	United States	Hispanics, African Americans, and Non-Hispanic Whites	Utilizing the social network theory as the conceptual framework, this study aims to examine effects of social support on receiving a mammogram among a representative sample of older adults, specifically African American and Hispanic populations in the United States. Logistic regression models were conducted using the 2008 and 2012 Health and Retirement Study data.	Findings from this study indicate that specific aspects of social support influence breast cancer screening participation among older Hispanic and non-Hispanic White women. However, this was not the case for the older Black women after adjusting for the sociodemographic factors. Findings can provide formative data to develop public health and social work interventions to increase positive social support and reduce negative social support by spouses and children to enhance breast cancer screening among older adults.	There may be additional factors that need to be considered within the context of social support, especially in light of the nonsignificant findings for Black women.
21. "This is some mess right here": Exploring interactions between Black sexual minority	Greene (2020)	Primary research	United States	Black sexual minority women	Participants were sampled nationally through social media, targeted emails, and referrals. Qualitative, in-depth interviews were conducted with 15 Black cisgender	Themes aligned with the SDT constructs of relatedness and autonomy. Some participants discussed feeling most	

women and health care providers for breast cancer screening and care					SMW, ages 38 to 64 years, who had a breast cancer diagnosis or recent abnormal mammogram. Interviews were conducted face-to-face or online, audio-recorded, and transcribed verbatim. Two analysts coded the interviews. Codes were analyzed across interviews to identify themes salient to SDT.	understood by Black and/or female providers who shared at least 1 of their identities. Feeling understood through shared identity contributed to participants feeling seen and heard by their providers. Participants who discussed negative experiences with providers believed that the provider made negative assumptions about them based on their race and/or sexual orientation.	
22. The Impact of Mammography Screening Guideline Changes Among Women Serving in the U.S. Military	Bytnar (2020)	Primary research	United States	Universally insured military servicewomen	This study evaluated the impact of the 2009 guideline changes among the population of universally insured military servicewomen, comparing the proportion of active duty women aged 40 to 64 receiving mammograms from fiscal years 2006 to 2015 using an interrupted time series analysis. Stratified analyses evaluated differences by age (aged 40-49, 50-64), race, military branch, and rank. This research is considered exempt by the Uniformed Services University Institutional Review Board.	The new guidelines recommended regular biennial screening for women beginning at age 50. The USPSTF guidelines had differential impacts among some subpopulations. While older women, aged 50 to 64 years, had a greater temporary reduction immediately after the guideline change, younger women aged 40 to 49 years had a longer-term reduction in screening following the guideline changes.	

						No racial disparities in the proportion screened or in the impact of the guideline change were noted in this population with universal health coverage.	
23. Health Beliefs and Breast Cancer Screening Practices Among African American Women in California	Davis (2021)	Primary research	California	Black women	Using a descriptive correlational design, a convenience sample of two hundred and eighty-two (n = 282) self-identified women from six regional chapters of a national Black women's political organization in California, completed a Demographic Data Questionnaire and Champion's Health Belief Model Scale which assessed the hypothesized relationships of health beliefs and breast cancer detection practices	Among this culturally diverse group of women (49.8% American, 28.8% African, 21.4% West Indian), health motivation was positively related to the practice of BSE and annual physician visitation for clinical breast examinations. Health locus of control was positively related to the practice of BSE. Having relatives and friends who were diagnosed with breast cancer was strongly associated with having a mammogram and annual physician visitation for clinical breast examinations.	
24. Effectiveness of Using Personal Health Records to Improve Recommended Breast Cancer Screening and Reduce Racial and	Kim (2020)	Primary research	United States	Women	The primary data used for this study were obtained from the 2015 Health Information National Trends Survey, and they were supplemented by the 2016 Area Health Resource Files. The study sample included women aged	We found a significant and positive association between the use of PHRs and recommended mammography use among women, with the likelihood of	

Geographic Disparities Among Women					40-75 years with no prior diagnosis of cancer. Because the use of PHRs as a key predictor of breast cancer screening may be endogenously determined, we used the instrumental variable (IV) approach to correct for estimation bias.	mammography screening increasing with more frequent use of PHRs. Furthermore, the effects of PHR use on mammography screening were found to be greater among Hispanic and Black women and those living in non-metropolitan areas compared with White women and those living in metropolitan areas, respectively. The use of PHRs for health information seeking can empower women at potential risk for breast cancer to participate in recommended screening mammography, particularly among those underserved and racial/ethnic minorities.	
25.Implementation of an integrated framework for a breast cancer screening and navigation program for women from underresourced communities	Henderson (2020)	Primary research	United States	Women	Using an integrated theoretical framework of the Practical, Robust Implementation and Sustainability Model and the Social Ecological Model, the University of Illinois Cancer Center and Mile Square Health Centers (MSHC) FQHC developed a breast	Increase in mammogram screening rates. Between January and December 2017, 103 women received a screening mammogram at MSHC. To increase screening rates, Mi-	

					cancer screening and navigation program, known as the Mile Square Accessible Mammogram Outreach and Engagement (Mi-MAMO) program, to tackle breast cancer disparities in Chicago among under resourced communities. To increase access to screening, patient navigators conducted community outreach activities. Partnerships were forged with community-based organizations, health care systems, and insurers. Outcomes were monitored with standardized performance measures. Intervention included Community Outreach Activities through Mile Square Accessible Mammogram Outreach and Engagement (Mi-MAMO) program: In-clinic navigation, Walk-In Wednesdays and mammogram parties, Mammogram days for insurers, Community outreach, Referrals	MAMO was started in August 2017. Between January and December 2018, the number of women who received a screening mammogram increased to 567. From August 2017 to December 2018, 779 women received navigation to screening and/or diagnostic services through the Mi-MAMO program. The majority of women were uninsured (63.9%), and 95.5% were racial/ethnic minorities. Twenty-four percent (n = 185) completed diagnostic services, and 10 women received positive breast cancer diagnoses (mean age, 49.7 years); all successfully navigated to treatment.	
26. Association of an Active Choice Intervention in the Electronic Health Record Directed to Medical Assistants With Clinician Ordering and	Hsiang (2019)	Primary research	United States	Women	A retrospective quality improvement study was conducted among 69 916 patients eligible for breast or colorectal cancer screening at 25 primary care practices at the University of Pennsylvania Health System	The sample eligible for breast cancer screening comprised 26 269 women with a mean (SD) age of 60.4 (6.9) years; 15 873 (60.4%) were white and 7715 (29.4%)	

Patient Completion of Breast and Colorectal Cancer Screening Tests					between September 1, 2014, and August 31, 2017. Data analysis was conducted from January 21 to July 8, 2019. From 2016 to 2017, 3 primary care practices at the University of Pennsylvania Health System implemented an active choice intervention in the electronic health record that prompted medical assistants to inform patients about cancer screening during check-in and template orders for clinicians to review during the visit. The primary outcome was clinician ordering of cancer screening tests. The secondary outcome was patient completion of cancer screening tests within 1 year of the primary care visit.	were black. For breast cancer screening, the intervention was associated with a significant increase in clinician ordering of tests (22.2 percentage points; 95% CI, 17.2-27.6 percentage points; $P < .001$) but no change in patient completion (0.1 percentage points; 95% CI, -4.0 to 4.3 percentage points; $P = .45$). An active choice intervention in the electronic health record directed to medical assistants was associated with a significant increase in clinician ordering of breast and colorectal cancer screening tests. However, it was not associated with a significant change in patient completion of either cancer screening test during a 1-year follow-up.	
27. Developing a Culturally and Linguistically Targeted Breast Cancer Educational Program for a	Fung (2021)	Primary research	NYC	African American, African-born, Chinese, Latina, and Muslim women, as well as	The Mount Sinai Mobile Breast Health Program in New York City collaborated with local organizations to develop culturally and linguistically appropriate breast cancer education programs aimed at increasing	Culturally and linguistically targeted education programs have the potential to increase screening mammography utilization in minority communities and to	Qualitative data gathered from focus groups conducted for the WP revealed major themes in Black women's beliefs and

Multicultural Population				LGBTQ individuals	screening mammogram utilization. Literature review of the barriers to mammography screening formed the basis to allow us to draft a narrative presentation for each targeted cultural group. The presentations were then tested with focus groups comprised of gatekeepers and members from local community and faith-based organizations which served the targeted populations. Feedback from focus groups and gatekeepers was incorporated into the presentations, and if necessary, the presentations were translated. Subsequently, the presentations were re-tested for appropriateness and reviewed for consistency in message, design, educational information, and slide sequencing.	reduce breast cancer disparities.	perceptions of breast cancer. The two prevailing themes were the sense of cancer fatalism—the belief that one is powerless in the face of cancer—and the stigmatization of cancer. Our presentation, therefore, sought to address breast cancer fatalism and stigmatization by providing breast cancer survival statistics and incorporating positive narratives from survivors. In particular, we emphasized that 99% of women diagnosed with localized (stages 0 or 1) breast cancer and treated live at least 5 years after diagnosis, underscoring the importance of early detection. Additionally, our presentation for Black women included a culturally targeted

							section on parity and breastfeeding in relation to breast cancer. Higher parity has been shown to be associated with increased risk of hormone receptor-negative and triple-negative breast cancers, which are disproportionately higher in Black women. However, breastfeeding appears to have protective effects against the aforementioned subtypes of breast cancer. Studies have found African-born women have limited knowledge and exposure to breast cancer screening information prior to their arrival in the USA. The lack of open breast cancer discourse and availability of prevention and treatment technologies in their country of

							origin has engendered the notion of breast cancer as “a white person’s disease”. Our presentation sought to clarify this misconception by providing noteworthy African breast cancer statistics. For example, we elucidated, “breast cancer is the most common type of cancer among African women”. We initially included country-specific incidence and mortality data, but community reviewers commented that the statistical information was unduly detailed for the purpose of the presentation. Consequently, we simplified the graphs and language to highlight the overall high incidence of breast cancer among African women and the disparities

							in screening between US-born and African-born women. Religious factors were also cited as barriers to breast cancer screening for African-born women. We addressed these beliefs in our presentation by explaining the basic etiology of cancer (i.e., “Cancer is the uncontrolled growth of abnormal cells in the body”) and by discussing its associated risk factors.
28. Factors Associated with Adherence to Preventive Breast Cancer Screenings among Middle-aged African American Women	Guo (2019)	Primary research	United States	African American women	This study is a longitudinal secondary data analysis of 3,911 African American participants of the Study of Women's Health Across the Nation. By using Systems Model of Clinical Preventive Care, multinomial logistic regression was applied to explore the likelihood of having breast cancer screenings (breast exam and/or mammogram) associating with predisposing factors, enabling factors,	Participants with older age, with higher education, having a healthcare provider for female health, in far distance, and with a cancer(s) were significantly more likely to adhere to the recommendations of breast cancer screenings. However, participants who did not have time to visit doctors, did not trust the physicians, and	

					referencing factors, and situational factors.	who smoked regularly were significantly less likely to adhere to the recommendations of breast cancer screenings.	
29. Examining Breast Cancer Screening Behavior Among Southern Black Women After the 2009 US Preventive Services Task Force Mammography Guideline Revisions	Farr (2020)	Primary research	United States	Southern Black women	The purpose of this study was to examine breast cancer screening behavior in a cohort of Southern Black women after the release of the 2009 USPSTF recommendations. Surveys assessing cancer screening information were collected from members of Black churches between 2006 and 2013. The sample was restricted to women aged 40 to 74 years, who did not report a breast cancer diagnosis, or a recent diagnostic mammogram (n = 789). Percentages of women ever completing a mammogram (age 40-49) and annual mammography (age 50-74) in 2006-2009 and 2010-2013 were compared using chi-square statistics. Logistic regression models were fit to determine the predictors of adherence to pre-2010 screening guidelines.	No significant changes in mammography rates were found for women in the 40-49 age group (X2 = 0.42, p = 0.52) nor for those in the 50-74 age group (X2 = 0.67, p = 0.41). Completing an annual clinical breast exam was a significant predictor of adherence to pre-2010 screening guidelines for both age groups (OR 19.86 and OR 33.27 respectively) and participation in education sessions (OR 4.26). Stability in mammography behavior may be a result of PCP's advice, or community activities grounded pre-2010 screening recommendations.	
30. Racial disparities in surveillance mammography among older	Teysir (2019)	Primary research	United States	Black and white women	Using the SEER-Medicare registry, we conducted an analysis of women ≥ 66 years diagnosed with early-stage (0-III) BC between 2000 and	We found that older black BC survivors continue to experience lower rates of surveillance	

breast cancer survivors					2011 who underwent BC surgery. The primary outcome was receipt of surveillance mammography within 12 months of surgery. Chi square analyses were used to compare characteristics between black and white women. Multivariate logistic regression was used to assess receipt of surveillance mammography after controlling for potential confounders.	mammography, even after adjusting for multiple potential confounders. There were 3353 black and 40,564 white women in the final cohort. After adjusting for confounders, black women were still 24% less likely than white women to receive surveillance mammography (Odds ratio 0.76, 95% CI 0.70-0.0.82). Those who were married, younger, in the highest income quartile, diagnosed at earlier stages, had a lower comorbidity score, or who resided in metropolitan areas were more likely to receive surveillance mammography ($p < 0.05$).	
31. Breast Screening Utilization and Cost Sharing Among Employed Insured Women Following the Affordable Care Act: Impact of Race and Income	Fazeli Dehkordy (2019)	Primary research	United States	Commercially insured women	We used Optum Clinformatics Data Mart deidentified patient-level analytic files between 2004 and 2014. We first visually inspected trends for screening mammography utilization and cost-sharing elimination over time by race and income. We then specifically calculated the slopes and compared trends before and after 2009 and	Impact of ACA cost-sharing elimination did not differ among various racial and income groups. Among our population of employer-based insured women, the racial gap in screening mammography use appeared to have closed and potentially	

					2010 to assess the impact of ACA implementation and USPSTF recommendation revisions on screening mammography cost-sharing elimination and utilization. All analyses were conducted in 2018.	reversed among African American women. Continued monitoring of screening utilization as health care policies and recommendations evolve is required, as these changes may affect race- and income-based disparities. A total of 1,763,959 commercially insured women, ages 40-74, were included. Comparing trends for cost-sharing elimination before and after the 2010 ACA implementation, a statistically significant but small upward trend was found among all races and income levels with no racial or income disparities evident. However, screening utilization plateaued or showed a significant decline after the 2009 USPSTF recommendation revision in all income and racial groups except for African Americans in whom screening rates	

						continued to increase after 2009.	
32. Regular Mammography Screening Among African American (AA) Women: Qualitative Application of the PEN-3 Framework	Adegboyega (2019)	Primary research	United States	African American (AA) women	As part of an intervention study, in-depth interviews were conducted with 39 AA women recruited from the emergency department of a public university hospital.	Women's perceptions included fear and limited knowledge. Enablers identified were cost, socioeconomic, and race-related discrimination, and health care previous experiences. Nurturers identified included observation of family experiences and lack of health-related social support.	
33. Gendered and racialized social expectations, barriers, and delayed breast cancer diagnosis	Kim (2018)	Primary research	Chicago	Women	Data from a randomized controlled trial, the Patient Navigation in Medically Underserved Areas study, were used. The likelihood of obtaining a follow-up screening mammogram was compared between women who identified ≥1 barriers and those who did not.	Of the 3754 women who received the Patient Navigation in Medically Underserved Areas navigation intervention, approximately 14% identified ≥ 1 barriers, which led to additional navigator contacts. Consequently, those women who reported barriers were more likely to obtain a subsequent screening mammogram. Black women, women living in poverty, and women with a higher level of distrust were less likely to report barriers. Minority women living	Black women were less likely to identify barriers compared with white and Hispanic women. Beyond economic difficulties, women often are caretakers and a source of social support for others, but because of their expected gender role, they may have difficulty expressing their own need for support.

						in poverty have always been the source of social support for others. However, gendered and racialized social expectations may affect the ways in which women seek help for their own health needs. A way to improve the effectiveness of navigation would be to recognize how minority women's gender images and expectations could shape how they seek help and support. A report of no barriers does not always translate into no problems.	
34. Affordable Care Act and Disparities in Health Services Utilization among Ethnic Minority Breast Cancer Survivors: Evidence from Longitudinal Medical Expenditure Panel Surveys 2008⁻2015	White-Means (2018)	Primary research	United States	non-Hispanic blacks (NHB), non-Hispanic whites (NHW) and Hispanics	This study draws on rich, nationally representative data, the 2008⁻2015 Medical Expenditure Panel Surveys, to estimate effects of the Affordable Care Act (ACA) on reducing disparities in and access to use of diagnostic and medical services for black and Hispanic breast cancer survivors. Random effects multinomial logit, flexible hurdle and Box-Cox estimation techniques are used.	The robust estimates indicate that the ACA narrowed the racial/ethnic disparity in health insurance coverage, health care utilization and out-of-pocket prescription drug expenditures among breast cancer survivors. Gaps in uninsurance significantly declined for black and Hispanic survivors.	Hispanic women generally and black breast cancer survivors specifically increased use of mammography services post-ACA.

35. Patient Voices Network: Bringing Breast Cancer Awareness and Action into Underserved Communities	Reilly (2018)	Primary research	United States	African American women	Concerned About You: Breast Cancer Awareness Walk & Wellness Event and its impact on an academic community partnership. A narrative approach was used. Meeting minutes and event planning notes were reviewed. Community participation rates and participant satisfaction were tracked using registration records and a survey administered at the event.	328 community members registered and 194 attended. Responses to a satisfaction survey indicated community buy-in and interest in future events. Two women were screened at the event and 78 were screened at a follow-up opportunity at their primary care practices. The process was driven by participatory guidelines and laid the foundation for future activities.	
36. Outcomes of a Community-based Breast Cancer Screening Program in Baltimore City	Ntiri (2018)	Primary research	Baltimore	uninsured African American women	A descriptive analysis of a community-based, cancer control program targeted at uninsured African Americans is presented. Program outcomes and correlates of program retention and BC detection are summarized.	Data for 5,669 enrollees and 10,357 mammograms were analyzed. Breast cancer was diagnosed in 113 women, 69% at an early stage. The majority (72%) of BC cases were diagnosed during the initial program cycle. The strongest correlates of program retention were non-Hispanic ethnicity and prior mammography (p<.0001). This community-based cancer control program provided an early BC detection	

						benefit to enrollees regardless of whether program services were conducted for only one cycle or were continued at regular intervals.	
37. Preferences for Communicating about Breast Cancer Screening Among Racially/Ethnically Diverse Older Women	Hoover (2019)	Primary research	Texas	Older women >= 70 years old and no history of breast cancer	In-depth interviews were conducted with 59 women with no breast cancer history. Non-proportional quota sampling ensured roughly equal numbers on age (70-74 years, ≥75 years), race/ethnicity (non-Hispanic/Latina White, non-Hispanic/Latina Black, Hispanic/Latina), and education (≤high school diploma, >high school diploma).	Thematic analyses revealed that rather than being told to get mammograms, participants wanted to hear about the benefits and harms of screening mammography, including overdiagnosis. Participants recommended that this information be communicated via physicians or other healthcare providers, included in brochures/pamphlets, and presented outside of clinical settings (e.g., in senior groups). Results were consistent regardless of participants' age, race/ethnicity, or education.	
38. Breast Cancer Screening for Patients of Rural Accountable Care Organization Clinics: A Multi-	Wang (2018)	Primary research	Nebraska	Women	Using medical record data of 8,347 women patients aged 50-74 years from eight rural ACO clinics in Nebraska, this study examined patient-, provider-, and county-level	The multi-level logistic regression results suggest that uninsured non-Hispanic Black patients were less likely to meet the	

Level Analysis of Barriers and Facilitators					barriers and facilitators for breast cancer screening. A generalized estimating equations model was used to account for the correlation among patients from the same provider and county.	biennial mammography screening guideline. Patients whose preferred language being English, having a preventive visit in the past 12 months, having one or more chronic conditions were more likely to meet the biennial mammography screening guideline. Patients with a primary care provider (PCP) that was male, without a medical doctor degree were less likely to screen biennially. Patients with a PCP that reviewed performance report quarterly, or manually checked patients' mammography screening status during visits were more likely to screen biennially. Interestingly, patients whose PCP reported being reminded by a care coordination team were less likely to screen biennially. Patients living in counties with more PCPs were also more	

						likely to screen biennially.	
39. Young Women's Perceptions Regarding Communication with Healthcare Providers About Breast Cancer, Risk, and Prevention	Lunsford (2018)	Primary research	United States	Young women	In 2015, 167 women aged 18-44 years participated in 20 focus groups segmented by geographic location, age, race/ethnicity, and family history of breast and ovarian cancer. Transcript data were analyzed using NVivo 10 software.	Although the majority of women talked to their doctor about breast and ovarian cancer, these conversations were brief and unsatisfying due to a lack of detail. Topics included family history, breast cancer screening, and breast self-examination. Some women with and without family history reported that healthcare providers offered screening and early detection advice based on their inquiries. However, few women took action or changed lifestyle behaviors with the intent to reduce risk as a result of the conversations.	
40. Effect of a Mammography Screening Decision Aid for Women 75 Years and Older: A Cluster Randomized Clinical Trial.	Schonberg (2020)	Primary research	United States	Women 75 years and older	A cluster randomized clinical trial with clinician as the unit of randomization. Intervention was: receipt of the DA (DA arm) or a home safety (HS) pamphlet (control arm) before a PCP visit. All analyses were completed on an intent-to-treat basis. The setting was 11 primary care practices in Massachusetts or North Carolina. Of 1247 eligible	Providing women 75 years and older with a mammography screening DA before a PCP visit helps them make more informed screening decisions and leads to fewer women choosing to be screened, suggesting that the DA may help reduce over-	

					women reached, 546 aged 75 to 89 years without breast cancer or dementia who had a mammogram within 24 months but not within 6 months and saw 1 of 137 clinicians (herein referred to as PCPs) from November 3, 2014, to January 26, 2017, participated. A research assistant (RA) administered a previsit questionnaire on each participant`s health, breast cancer risk factors, sociodemographic characteristics, and screening intentions. After the visit, the RA administered a postvisit questionnaire on screening intentions and knowledge. The primary outcome was receipt of mammography screening within 18 months.	screening. Participants were followed up for 18 months for receipt of mammography screening (primary outcome). To examine the effects of the DA, marginal logistic regression models were fit using generalized estimating equations to allow for clustering by PCP. Adjusted probabilities and risk differences were estimated to account for clustering by PCP. Results: Of 546 women in the study, 283 (51.8%) received the DA. Patients in each arm were well matched; their mean (SD) age was 79.8 (3.7) years, 428 (78.4%) were non-Hispanic white, 321 (of 543 [59.1%]) had completed college, and 192 (35.2%) had less than a 10-year life expectancy. After 18 months, 9.1% (95% CI, 1.2%-16.9%) fewer women in the DA arm than in the control arm had undergone mammography screening (51.3% vs	

						60.4%; adjusted risk ratio, 0.84; 95% CI, 0.75-0.95; P = .006). Women in the DA arm were more likely than those in the control arm to rate their screening intentions lower from previsit to postvisit (69 of 283 [adjusted %, 24.5%] vs 47 of 263 [adjusted %, 15.3%]), to be more knowledgeable about the benefits and harms of screening (86 [adjusted %, 25.5%] vs 32 [adjusted %, 11.7%]), and to have a documented discussion about mammography with their PCP (146 [adjusted %, 47.4%] vs 111 [adjusted %, 38.9%]). Almost all women in the DA arm (94.9%) would recommend the DA.	
41. A lay health worker intervention to improve breast and cervical cancer screening among Latinas in El Paso, Texas: A randomized control trial	Savas (2021)	Primary research	Texas	Low-income, primarily uninsured Latina	In 2015, Breast and Cervical Cancer Screening (BCCS) program promotoras recruited Latinas overdue for breast and/or cervical cancer screening in community settings. Promotoras consented eligible women and conducted baseline surveys before individually randomizing women into	At follow-up, among women in need of breast cancer screening, those in the intervention group were significantly more likely to complete a mammogram than those in the control group (PP: 53.4% vs.	

					control (n = 313) or intervention (n = 314) groups. Control participants received printed material providing basic information about breast and cervical cancer screening. Intervention participants received promotora-delivered one-on-one breast and cervical cancer screening education followed by navigation calls, providing assistance to address personal and logistic barriers to accessing clinical services. They assessed breast and cervical cancer screening outcomes using a 6-month follow-up survey. Per protocol (PP) and intent to treat (ITT) analyses are reported.	40.1%, p = .013; ITT: 47.9% vs. 35.2%, p = .011).	

Appendix F. Template Analysis: Summary Matrix

Motivation to screen	Engagement w/ chatbot	Reactions to media	Reactions to content	Perception of chatbot	Comfortability	Trust	Usefulness	Relatability	Desired content	Desired features/ function	Usability
Motivation to screen – being there for family/ living your life (1)<br>Emphasize learning self-love and care (2)<br>Need to overcome	Groups disagreed on whether screens were engaging(1,2,3)<br>Discussed social knowledge/ awareness(1)	*video*<br><br>Overall positive reactions to videos (1,2,3)<br><br>Having a BC survivor answer questions	Liked including discussion of cost(1,3)<br><br>Did not like the word dis-proportionately– it's unclear(1)<br><br>Did not like: "can you tell	Differing reactions to name – multiple groups asked whether acronym (1,2)	Majority of participants thought they would feel comfortable using chatbot although some skepticism	Multiple participants did not trust Sesi citing name and use of AI ["got a problem with that whole Big Brother	Participants thought that they would use at least some of information provided by chatbot (2,3)<br><br>Saw chatbot as particularly useful for younger	Participants disagreed whether chatbot persona was relatable (1,2)<br><br>Concern about cultural	Increase social acceptability of talking about health (1)<br><br>Use chatbot to increase sociability of	Reminders for appts once scheduled (2,3)<br><br>Agreed like to request female technician (1,2)	Seemed usable based on screenshots (1)<br><br>Hard to assess usability from screenshot

competing priorities(1, 2)<br>Discussed experience of racism as barrier(2)	Discussed the challenge that everyone will want to receive info in the same way(1)	gives credibility (2)<br>Thought content was appropriate<br>Liked conversation about cost (2)<br>Recommended adding topics [eg, prepare people for discomfort(2); more info about benefits(3); reassurance of follow up if abnormal result(2)]<br>Thought interviewer should identify as a Black woman eligible for breast cancer screening<br>*Images*<br>Liked showing images of mammography machine – helped to know what to expect	me what prevented you from attending the appointment" (seemed judgmental)(1,2)<br>"based on records" felt like privacy invasion(2)<br>Initial messaging was not clear about purpose(2)<br>Easy to understand (3)	Welcoming, positive, warm, nice image, nice smile (1)<br>Participants within groups disagreed about reaction to persona – both positive and negative<br>(skeptical) (2,3)	about using AI. (1,3)	thing."(3)] (2,3)<br>Not sure whether trustworthy source of information (3)<br>Did not think should be explicitly linked to university(2)<br>One participant felt ok answering a few questions and then having further discussion with PCP(1)	women/ first time screening(1,2,3)	inclusiveness – felt that it wasn't personalized outside of community partner involvement (2,3)<br>"And it just didn't speak to me as being a Black woman. That's what I'm going to say. But, you know, let's just be honest. Who made the app?"(2)<br>"Sometimes, because we're black, other communities patronize on us being black to... they just patronize us as if we know what it is to be in Africa and we don't. We've	screening (1)<br>More information about self-exams (1,2,3)<br>BC data about Black women specifically ["I used to believe that certain diseases were only for white people"(2)] (2,3)<br>Frequency of screening(2)<br>Emphasize importance of BCS even if haven't felt a lump (3)<br>information re: how to prepare, eg, no lotion or deodorant, wear pants (prep to undress from waist up); when to arrive and how	Liked scheduling (2)<br>Agreed would like to use to make connections "mammogram parties" (1,2)<br>Participants thought that it would be helpful to be able to use the chatbot at different points in time –may have questions that they thought of later (1)<br>Generally positive to sharing quotes of other women's experiences (1)<br>Some way to refer to PCP when they have questions not answered by chatbot ["you can't	s (without actually testing) (1)<br>Questioned usability as app – thought it would take too long (2,3)<br>Emphasized efficiency which could be quicker than phone call: receive message from chatbot and schedule mammogram in < 15 min (2,3)

		Liked that both tech and patient were Black women [“It's like, "Oh, that looks like me. Oh, that looks like somebody I can relate to."(1)] (1,2,3)<br><br>in videos, what to expect, participants discussed getting letters rather than phone calls about results – make sure this information is accurate and represents what will actually happen (3)						never been to Africa. We still have the same issues, yes, but we've never been there so we can't relate to certain things or cultures that have because we don't have that. We've never, that was not brought along with us here.”(3)	long it will take; what to expect/ next steps after mammogram (1,3)	really ask what you really want to ask”] (3) A “back” button or way to correct if mistake made (3)	
