# Supplementary material for: Leveraging human-centered design and causal pathway diagramming toward enhanced specification and development of innovative implementation strategies: a case example of an outreach tool to address racial inequities in breast cancer screening
Source: Implement Sci Commun. 2024 Mar 28;5:31. doi: 10.1186/s43058-024-00569-w (PMC10976783; doi:10.1186/s43058-024-00569-w)
Supplement: Supplementary file 2 — Additional file 2: Fig. S1. Early Chatbot Prototype. [file 43058_2024_569_MOESM2_ESM.docx]

**Figure S1. Early Prototype**


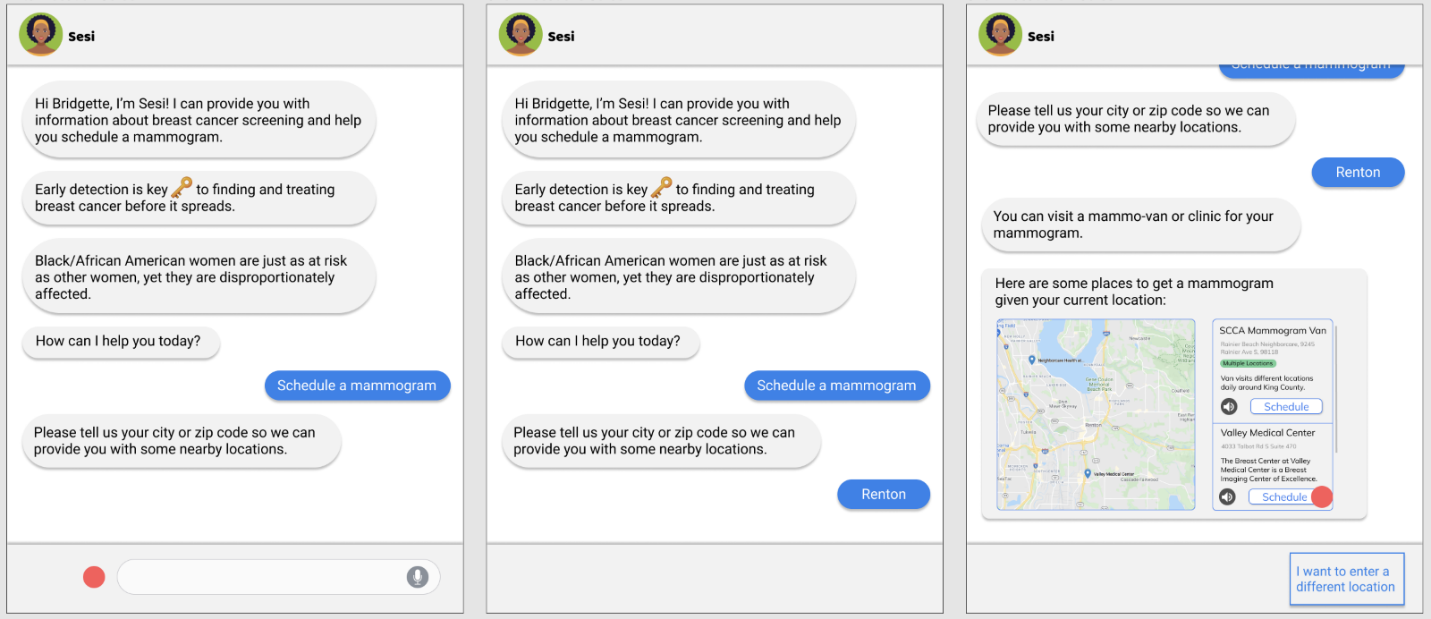


Avatar attribution: Vecteezy. Beautiful black woman avatar character icon Free Vector. https://www.vecteezy.com/vector-art/2002247-beautiful-black-woman-avatar-character-icon. Accessed on February 24, 2024.
